# Division-Based, Growth Rate Diversity in Bacteria

**DOI:** 10.3389/fmicb.2018.00849

**Published:** 2018-05-11

**Authors:** Ghislain Y. Gangwe Nana, Camille Ripoll, Armelle Cabin-Flaman, David Gibouin, Anthony Delaune, Laurent Janniere, Gerard Grancher, Gaelle Chagny, Corinne Loutelier-Bourhis, Esther Lentzen, Patrick Grysan, Jean-Nicolas Audinot, Vic Norris

**Affiliations:** ^1^Laboratory of Microbiology Signals and Microenvironment, Department of Biology, University of Rouen, Mont Saint Aignan, France; ^2^Department of Biology, University of Rouen, Mont Saint Aignan, France; ^3^Groupe de Physique des Matériaux, Centre National de la Recherche Scientifique, Département de Biologie, Université de Rouen Normandie, Saint-Etienne du Rouvray, France; ^4^Laboratoire iSSB, Evry, France; ^5^R. Salem Laboratory of Maths, UMR 6085 Centre National de la Recherche Scientifique-University of Rouen, Saint Etienne du Rouvray, France; ^6^UMR Centre National de la Recherche Scientifique, 6014 COBRA, University of Rouen, Mont Saint Aignan, France; ^7^Material Research & Technology Department, Luxembourg Institute of Science and Technology, Belvaux, Luxembourg

**Keywords:** heterogeneity, asymmetry, bacteria, DNA segregation, cell cycle, secondary Ion mass spectrometry, NanoSIMS 50, isotope-labeling

## Abstract

To investigate the nature and origins of growth rate diversity in bacteria, we grew *Escherichia coli* and *Bacillus subtilis* in liquid minimal media and, after different periods of ^15^N-labeling, analyzed and imaged isotope distributions in individual cells with *Secondary Ion Mass Spectrometry*. We find a striking inter- and intra-cellular diversity, even in steady state growth. This is consistent with the strand-dependent, hyperstructure-based hypothesis that a major function of the cell cycle is to generate coherent, growth rate diversity via the semi-conservative pattern of inheritance of strands of DNA and associated macromolecular assemblies. We also propose quantitative, general, measures of growth rate diversity for studies of cell physiology that include antibiotic resistance.

## Introduction

Although phenotypic diversity is fundamental to the way populations of bacteria deal with the opportunities and risks presented by their environment including exposure to antibiotics (Smits et al., 2006; Maisonneuve and Gerdes, 2014), the extent of this diversity and the mechanisms responsible for generating it remain to be fully elucidated. Phenotypic diversity is reflected in the diversity of growth rates. The conclusion drawn from early, influential studies on non-differentiating bacteria growing on nutrient plates (Schaechter et al., 1962) or in liquid media (Ecker and Kokaisl, 1969) was that individual cells had the same growth rate. In recent years, however, the results from microfluidics and other studies have pointed to the radically different conclusion that individual bacteria grow in apparently the same conditions with different rates (Godin et al., 2010; Campos et al., 2014; Taheri-Araghi et al., 2015; Wallden et al., 2016). That said, there is no disagreement that major changes in the growth rate *at the level of the population* due to growth in nutritionally different media are accompanied by major changes in the size and composition of the cells (Schaechter et al., 1958).

Growth rates affect the key steps of the cell cycle (Weart et al., 2007) and, reciprocally, the cell cycle affects growth rates (Wang et al., 2010; Bowman et al., 2013; Osella et al., 2014). If an evolutionarily useful phenotypic diversity at the level of growth rates is to be generated—for example, to allow some bacteria to escape the action of an antibiotic (Balaban et al., 2004; Kim and Wood, 2016)—each phenotype must be coherent with respect to the set of genes expressed (Norris and Amar, 2012). To achieve such coherent diversity, we have proposed that one of the parental strands of DNA can be physically associated with proteins appropriate for a survival strategy whilst the other strand can be physically associated with proteins appropriate for a growth strategy, so allowing division to generate daughters with different, coherent phenotypes (Rocha et al., 2003). To investigate growth rate diversity, we grew the model organisms *Escherichia coli* and *Bacillus subtilis* in liquid minimal media, labeled them with the rare, stable isotope, ^15^N, and analyzed them using the sensitive, quantitative imaging technique of Secondary Ion Mass Spectrometry (SIMS) (Musat et al., 2008; Boxer et al., 2009; Petroff et al., 2011).

## Materials and methods

### Cell culture

*E. coli* BL21 (B F^−^ *ompT lon hsdS*(*rB*^−^ *mB*^−^) *gal dcm* λ(DE3) was grown continuously at 37°C in M9 medium containing per liter 0.1 mmol CaCl_2_, 8.498 g Na_2_HPO_4_-2H_2_O, 3 g KH_2_PO_4_, 1 g NH_4_Cl, 2 mmol MgSO_4_, 0.5 g NaCl, 4 g D-glucose. Twenty-five milliliters of culture was shaken at 240 r.p.m. in a 250 ml Ehrlenmeyer flask in either a Buhler incubator or a New Brunswick G76 shaker. The mass doubling time was 64 min (OD_600_). After 15 generations at an OD_600_ that never exceeded 0.1, bacteria were inoculated, via preheated pipettes, at the dilution of 1:10 in new M9 medium with ^15^NH_4_Cl (98% ^15^N, ISOTEC, USA) as the only nitrogen source (note that an OD_600_ of 0.1 corresponds to early exponential growth and, in the conditions we used, plenty of nutrients are available and the cells could have continued growing exponentially for several more generations). One milliliter samples were then taken at 2, 4, 8, 16, 32, 64, and 128 min; the OD_600_ was <0.05 after labeling for 128 min. Growth was stopped by adding 1 mL of M9 ^14^N medium at 0°C. All subsequent manipulations were then performed at or below 4°C. To exclude the artifactual incorporation of isotopes during this cold treatment, cells were grown in ^14^N medium and growth was stopped by adding M9 ^15^N medium at 0°C and cells were prepared and analyzed as described below; no incorporation of ^15^N was detected (Supplementary Figure 1). To exclude the presence of contaminant bacteria, a standard metabolic test, API, was performed, which confirmed that the bacteria were *E. coli* (not shown); moreover, the bacteria produced identical colonies on agar plates (not shown) and the mass doubling time as measured by optical density corresponded to that previously reported for this strain in the same growth conditions. Finally, if it is supposed we started with equal numbers of two different species, for example, one with a mass doubling time of 36 min and the other 72 min; after 960 min of steady-state growth—round 15 generations—the slower species would be present at a frequency of 2^960/72^/2^960/36^ so around 1/10000, effectively leaving a single species growing at the faster rate.

*B. subtilis 168 trpC2* (Burkholder and Giles, 1947) was grown at 40°C in Spizizen medium containing per liter 50 mmol CaCl_2_, 14 g K_2_HPO_4_, 6 g KH_2_PO_4_, 2 g (NH_4_)_2_SO_4_, 1 g C_6_H_5_Na_3_O_7_.2H_2_O, 2 mmol MgSO_4_, 11 mg Fe III citrate, 10 μmol MnCl_2_, 1 μmol FeSO_4_, 4 mg FeCl_3_, 2g D-glucose, 100 mg tryptophan, and 1 g casein hydrolysate. 50 ml of culture were shaken at 240 r.p.m. in a 250 ml Ehrlenmeyer flask in a Buhler incubator. After a 1:50 dilution of an overnight culture, bacteria were grown for 3 h to reach exponential phase (in which the mass doubling time was measured at OD_600_ as 42 min). At the start of this exponential phase, ^13^C_6_-D-glucose (99% ^13^C, ISOTEC, USA) and ^15^NH_4_Cl (98% ^15^N, ISOTEC, USA) were added to give final ratios of ^13^C-D-glucose:^12^C-D-glucose of 1 and ^15^NH_4_Cl:^14^NH_4_Cl of 1. Samples of 10 mL were then taken at 90 min and 120 min. Growth was stopped by adding 10 mL of Spizizen medium at 0°C. All subsequent manipulations were then performed at or below 4°C. Samples were centrifuged in a Sigma 3K18C at 6000 r.p.m. (8,700 g) for 10 min and the pellets were resuspended in 0.1 mol/L cacodylate plus 0.04% MgCl_2_ (to help avoid autolysis), then centrifuged again at 6000 r.p.m. for 15 min.

### Preparation of cells

**Figure 1:**
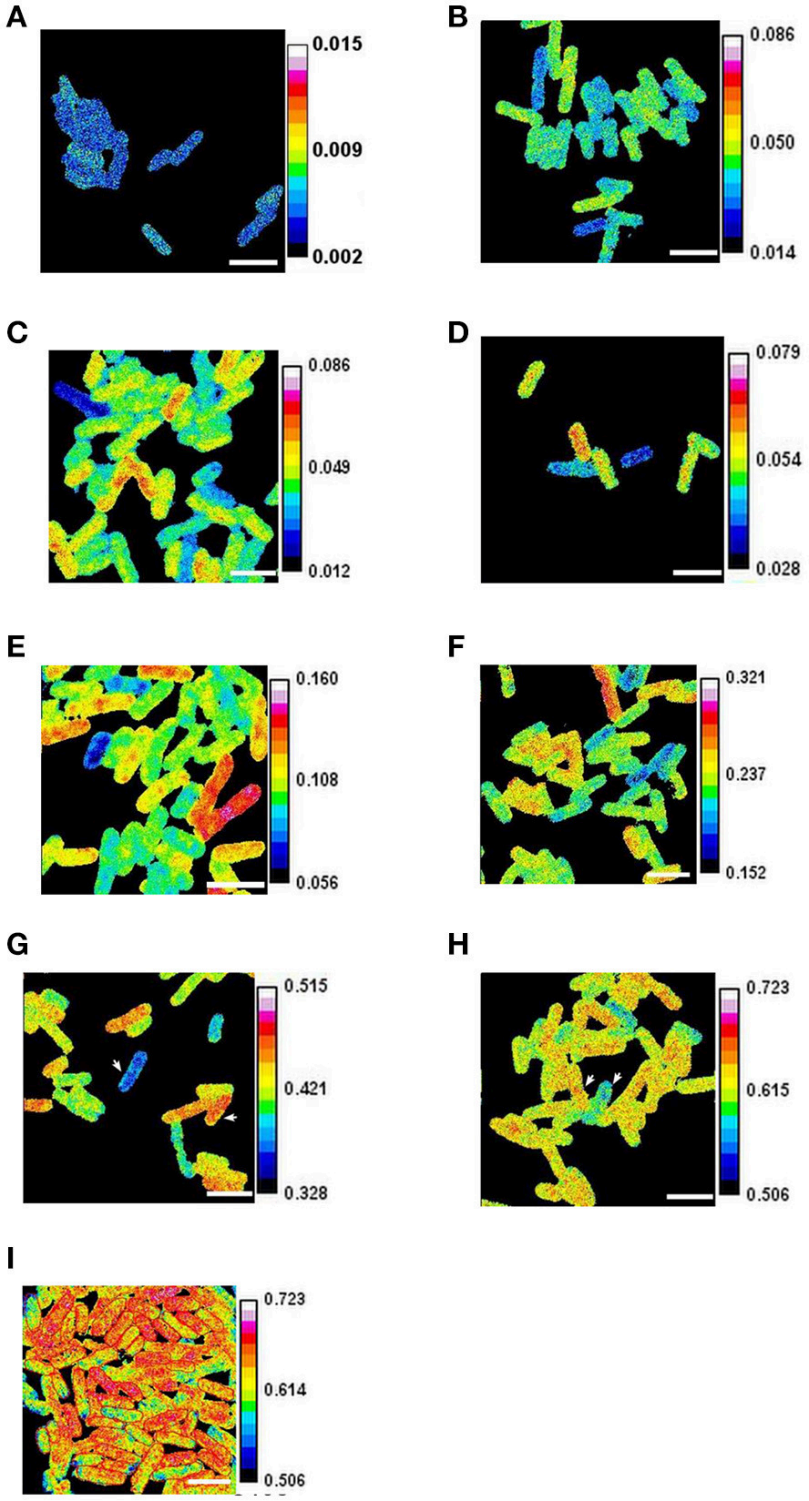
Heterogeneous growth of *E. coli*. Cells growing in steady state (after 15 generations of growth at an OD600 below 0.1) were labeled with 90% ^15^N for periods of **(A)** 0 min. **(B)** 2 min. **(C)** 4 min **(D)** 8 min. **(E)** 16 min. **(F)** 32 min. **(G)** 64 min. **(H)** and **(I)** 128 min. In **(I)** the cells were fixed with formaldehyde and some outlined for analysis. The ratios of ^12^C^15^N/(^12^C^14^N+^12^C^15^N) were obtained with a NanoSIMS 50. The arrowheads show cells with mass doubling times estimated using isotope incorporation as 61 min and 82 min in **(G)**, and as 70 min and 80 min in **(H)**. Scale bars 2 μm.

Samples were centrifuged in a Sigma 3K18C at 6000 r.p.m. (8,700g) for 10 min. Then one of two fixation methods was used. Cells were fixed either with ethanol but without formaldehyde (in the case of all Figures shown except Figure 1I and Supplementary Figure 2G) or with formaldehyde but without ethanol (in the case of Figure 1I and Supplementary Figure 2G):

Method 1: The pellets were resuspended in 0.2 mol/L cacodylate plus 0.04% MgCl_2_ (to help avoid autolysis), then centrifuged again at 6000 r.p.m. for 15 min. The following dehydration step was performed at 4°C for both *B. subtilis* and *E. coli*. The pellet was resuspended in 25% ethanol (in 0.2 mol/L cacodylate) for 20 min, then 50% ethanol (in 0.2 mol/L cacodylate pH 7.4) for 15 min and centrifuged at 6,000 r.p.m. (8700*g*) for 10 min. The pellet was then suspended in 10 μL of 50% ethanol (in 0.2 mol/L cacodylate) and 2 μL were deposited on a silicon chip (which had previously been cleaned by sonication during successive immersions in distilled water, 96% ethanol, acetone, 3:1 H_2_SO_4_:H_2_O_2_, water, and finally 96% ethanol). The dehydrated bacteria on the chip were then baked in a vacuum at 50°C for *B. subtilis* and at 40°C for *E. coli*.

Method 2: The pellets were resuspended in 1 mL of formaldehyde 3.7% buffered with 0.2 mol/L cacodylate pH 7.4 for 30 min, then centrifuged again at 12,000*g* for 10 min at 4°C. The pellet was rinsed for 10 min in 0.2 mol/L cacodylate pH 7.4; this rinsing was then repeated once. The pellet was then suspended in 20 μL of 0.2 mol/L cacodylate and 2 μL were deposited on a silicon chip. The dehydrated bacteria on the chip were then baked in a vacuum at 50°C.

### SIMS analysis

The bacteria on the chip were analyzed using a Zeiss Scanning Electron Microscope (which showed the bacterial morphology expected with no evidence for lysis, Supplementary Figure 3) and a CAMECA NanoSIMS 50. The latter entails bombardment of the sample with a high energy primary beam of ions that is focused on the surface and rastered across it; this fragments molecules contained in a nanovolume and sputters them out; a proportion of these fragments is collected as secondary ions and counted with a mass spectrometer (the useful yield), hence the name *Secondary Ion Mass Spectrometry*. These ions counted in each nanovolume can then be displayed as an image. In our conditions, the chip was bombarded with a primary beam of Cs^+^ ions (the impact energy for the primary ions was set to 16 kV and the primary current to 1.3 pA or to 0.8 pA for the *B. subtilis* and *E. coli* experiments, respectively), the mass spectrometer set at a mass resolution of 5000 = M/ΔM (where ΔM is the difference between the masses of two secondary ions with the same mass number, M); five out of the six following secondary ions were counted simultaneously from the same place on the surface of the sample: ^12^C, ^13^C, ^12^C^14^N, ^12^C^15^N or ^13^C^14^N, and ^13^C^15^N.

In a “sputtered section,” all material containing carbon and nitrogen is sputtered out and a proportion then detected (the useful yield); the labeled nitrogen is sufficient to allow detection of membrane and cytoplasm which both contain this element. Three successive sections of the cells were sputtered out in our conditions and all the counts in the middle section were used for imaging in all the figures shown except for Supplementary Figures 4, 5. Assuming that the density of the sample is 5.10^22^ atoms/cm^3^ and that the sputtering yield (the number of sputtered particles per incident primary ion) was in the range 2 to 10, about 5 nm of material was sputtered out per section of *B. subtilis* (1.3 pA, raster 8 μm, pixels 128, dwell time 40 ms/px) and about 5 nm per section of *E. coli* (0.8 pA, raster 10 μm, pixels 256, dwell time 10 ms/px). ImageJ was then used for the extraction, treatment and assembly of the data (Mutterer and Zinck, 2013). Excel and OpenOffice were also used for data collection and statistical analysis. The naturally occurring ratios of ^15^N:^14^N and ^13^C:^12^C are 0.0036 and 0.011, respectively. Isotope fractions such ^12^C^15^N/(^12^C^14^N+^12^C^15^N) were used to both estimate mass doubling times (as equaling ^15^N/(^14^N+^15^N), see below) and avoid artifacts due to matrix effects (Lhuissier et al., 2000; Peteranderl and Lechene, 2004); the latter arise because of different useful yields in different matrices and can be allowed for by using the fraction ^12^C^15^N/(^12^C^14^N+^12^C^15^N) because both ions are affected in the same way.

The mean values of ^13^C^15^N/(^13^C^15^N+^12^C^15^N) obtained for *E. coli*, 0.0112 ± 0.0003 after 128 min, were the same as the natural fraction of ^13^C, as expected. To ensure the validity of our results, we excluded those images in the *E. coli* experiments that had a bacterium with a mean value of ^13^C^15^N/(^13^C^15^N+^12^C^15^N) >0.016 or <0.006, that is, those bacteria with fractions out of the range 0.011 ± 0.005. This choice was because 0.005 is the standard deviation typically obtained from the pixels of a single bacterium.

Instrumental isotope fractionation in SIMS might be considered a potential problem. This fractionation means that, globally, two different numbers of secondary ions are counted for the same number of atoms of two different isotopes of the same element present in the sample (so that, for example, the isotope ratio of ^12^C^15^N/(^12^C^14^N+^12^C^15^N) counted by the machine differs from the actual ratio). The reason this might be a problem is because the fractionating effects during ionization and analysis are likely to contribute to the isotope ratios on which our conclusions are based. However, a biological variability in a reference sample (a single cell with the normal isotope ratio) has been reported as being <2% (Kopf et al., 2015). Hence the differences between the real and the counted numbers of isotopes are small compared with (1) the differences due to the Poisson nature of the counting process [the percentage error = 100/(square root of the mean)] which in the case of the 32 min and 128 min labeling, for example, is typically 7 and 3%, respectively, on the same image), (2) the differences between the bacteria (in the text we report that the %CV ranges from 5 to 20) and (3) the level of diversity apparent even within an image, which is acquired with the same settings within a short period of time; admittedly, the counting artifact could become more significant when comparing acquisitions obtained at different times and on different samples. However, it should be noted that the diversity we describe below is apparent even within a single image. And even if there were a significant error in our fractionation, this would apply to all the bacteria and simply shift the mean of the distribution of isotope ratios without affecting its variance (i.e., the growth rate diversity we observe would still be true); this is because there is a linear relationship between the isotope ratio of ^12^C^15^N/(^12^C^14^N+^12^C^15^N) measured by SIMS and bulk isotope ratios measured by other techniques (see Table 1 and Figure C8 in Kopf et al., 2015). Finally, our estimation of the mass doubling time of the population based on the isotope ratios matches the estimation of the mass doubling time based on optical density, which it would not do if there were a significant error in our measurements.

### Mass spectrometry

Electrospray ionization-mass spectrometry (ESI-MS) experiments were performed using a Bruker HCT Ultra ETD II quadrupole ion-trap (QIT) equiped with an ESI source, Esquire control 6.2 and Data Analysis 4.0 software package (Bruker, Bremen, Germany). The ESI parameters were capillary and end plate voltages set respectively to +3.5 and +3.0 kV in negative ion mode. The skimmer and the capillary exit voltages were set respectively to −30 and −80V and the injection low mass cut-off (LMCO, corresponding to the “trap drive” parameter) value was *m/z* 40. Sample solutions were either infused into the ESI source at a flow-rate of 3 μL.min^−1^ by means of a syringe pump (Cole-Palmer, Vernon Hills, Illinois, USA) for full scan data or injected into the ESI source at a flow-rate of 100 μL.min^−1^ by means of a liquid chromatographic (LC) system (Agilent 1200 chromatographic system equipped with a G1379B degasser, a G1312A high pressure binary pump and a G1329A autosampler, Agilent technologies, Waghaeusel-Wiesental, Germany) for full scan data as well for the more specific precursor-to-product ion transition termed selected reaction monitoring (SRM). For infusion, the nebulizer gas (N_2_) pressure, drying gas (N_2_) flow rate and drying gas temperature were 10 psi, 7.0 L/min and 300°C, respectively. For LC-injection, the nebulizer gas (N_2_) pressure, drying gas (N_2_) flow rate and drying gas temperature were 30 psi, 10.0 L/min and 300°C, respectively. Helium pressure in the ion trap was 1.1 × 10^−5^ mbar. Full scan spectra were acquired in the *m/z* 50–1,000 range, using a scan rate of 8100 *m/z* per second (“Standard-Enhanced” mode). The number of ions entering the ion trap was automatically adjusted by controlling the accumulation time with the ion charge control (ICC) mode (target 50,000) with a maximum accumulation time of 50 ms. The values of spectra averages and rolling average were 5 and 2 respectively. The selected reaction monitoring (SRM) experiments using glutamate specific precursor-to-product ion transition “*m/z* 146 → *m/z* 128” were carried out by collision induced dissociation (CID) using a resonant excitation frequency with ramping amplitude from 0.06 to 0.42 V_p−p_, helium as the collision gas, isolation width of 1 *m/z* unit for the precursor ion (*m/z* 146) and 1 *m/z* unit for the product ion (*m/z* 128).

### Estimation of population growth rate

We took growth to be the increase in cell mass (irrespective of its molecular nature) as determined by nitrogen uptake thereby disregarding the question of whether cells have significantly different densities (Poole, 1977; Martinez-Salas et al., 1981; Makinoshima et al., 2003). After addition of ^15^N to the *E. coli* culture, we measured the isotope fraction of nitrogen, x(t), using ^12^C^15^N/(^12^C^14^N+^12^C^15^N) after different periods, t. Two independent series of experiments, A and B, were performed and from 2 to 11 images were acquired and analyzed after each period of labeling. The values at 4 min for one of the B series were discarded (see SIMS analysis above). A total of 2174 bacteria in 83 images were analyzed. Given that an unsynchronized population of bacteria grows exponentially:

(1) $$\begin{array}{l} \text{x}\left(\text{t}\right)=\left\{{\text{x}}_{\text{ext}}\times \left(2^{\text{t}/\text{g}}-1\right)\;+\;x_{0}\right\}/2^{\text{t}/\text{g}} \end{array}$$

Where t is the period of labeling, g is the mass doubling time, x_0_ is the natural level of ^15^N (0.00366), and x_ext_ is the fraction of ^15^N to total N in the final medium after addition of the ^15^N. x_ext_ is obtained from adding 9 volumes of 98% ^15^N (as estimated by the supplier) to 1 volume of natural nitrogen hence x_ext_ = (0.9 × 0.98) + (0.1 × 0.00366) = 0.882

To be coherent with the model of exponential growth of a population of bacteria where it is the population mass which is considered, all the values of the x(t) measured at each pixel of all the bacteria visible in a given image j were pooled and the mean of the isotope fractions of the pixels in an image, $\bar{x_{j}\left(t\right)}$, and standard deviations were calculated. In exponential growth where γ = (ln2)/g, the growth of the population can be expressed as:

(2) $$\begin{array}{l} \text{Y}\left(\text{t}\right)=\left({\text{x}}_{\text{ext}}-{\text{x}}_{0}\right)/\left({\text{x}}_{\text{ext}}-\text{x}\left(\text{t}\right)\right)=\exp\left(\gamma \text{t}\right)=2^{\text{t}/\text{g}} \end{array}$$

Hence, this can be expressed in terms of the image means:

(3) $$\begin{array}{l} \bar{{\text{Y}}_{\text{j}}\left(\text{t}\right)}=\left({\text{x}}_{\text{ext}}-{\text{x}}_{0}\right)/\left({\text{x}}_{\text{ext}}-\bar{{\text{x}}_{\text{j}}\left(\text{t}\right)}\right)\;=\exp\left(\gamma \text{t}\right) \end{array}$$

Supplementary Figure 6 shows that all the image means $\bar{{\text{Y}}_{\text{j}}\left(\text{t}\right)}$, are well fitted, as expected, by an exponential function a × exp(bt) with a determination coefficient of 0.9903 and agreement with equation (3) since a = 0.9938 (close to 1) and γ = b = 0.0106 min^−1^ corresponds to a population doubling time of ln(2)/0.0106 = 65 min whilst the value obtained using OD_600_ was 64 min. Supplementary Figures 7, 8 show other representations of these results highlighting perturbations of growth at short labeling periods even though these perturbations do not affect cell division (Supplementary Figure 9).

### Estimation of growth rates of individual cells

The nature of the growth of individual bacteria has a long history. For many years, there was a controversy over whether the growth of individual bacteria in steady state conditions should be described by an exponential or by some other function (Kubitschek, 1986; Cooper, 2006). It is now generally believed that individual bacteria grow exponentially. This belief is supported by the observations using optical microscopy of bacteria growing on surfaces or in channels (Wang et al., 2010; Osella et al., 2014) and, with less certainty, by observations based on the buoyant density of bacteria (Godin et al., 2010).

Our estimation of the mass doubling times of individual cells using ^15^N incorporation assumes the exponential growth of the individual bacterium, the conservation of nitrogen, the relatively slight effect of differential segregation of ^14^N and ^15^N (see below) and no significant changes in the media during the experiment. To grasp rapidly the rationale for our estimation, imagine that all of the unlabeled material in a new-born cell at the start of the labeling period remains within that cell during the labeling whilst all the material labeled during the period goes into the other cells. The ratio of unlabeled to labeled material is therefore 1:1 after 1 mass doubling time in the label, 1:3 after 2 mass doubling times etc. Hence:

(S1) $$\begin{array}{l} \text{x}\left(\text{t}\right)=\left\{{\text{x}}_{\text{ext}}\times \left(2^{\text{t}/\text{g}}-1\right)+{\text{x}}_{0}\right\}/2^{\text{t}/\text{g}} \end{array}$$

Where x_ext_ = the fraction of ^15^N to total N in the final medium after addition of the ^15^N, x_0_ = the natural fraction of ^15^N, t is the period of labeling, and g = the mass doubling time.

(S2) $$\begin{array}{lll} \text{x}\left(\text{t}\right) & = & \left[{\text{x}}_{0}+\left\{{\text{x}}_{\text{ext}}\times \left(2^{\text{t}/\text{g}}-1\right)\right\}\right]/2^{\text{t}/\text{g}} \end{array}$$

(S3) $$\begin{array}{lll} 2^{\text{t}/\text{g}} & = & \left\{{\text{x}}_{0}/{\text{x}\left(\text{t}\right)-\text{x}}_{\text{ext}}/\text{x}\left(\text{t}\right)\right\}/\left[1-\left({\text{x}}_{\text{ext}}/\text{x}\left(\text{t}\right)\right)\right] \end{array}$$

(S4) $$\begin{array}{lll} \ln\;2^{\text{t}/\text{g}} & = & \ln\left\{\left({\text{x}}_{0}/{\text{x}\left(\text{t}\right)-\text{x}}_{\text{ext}}/\text{x}\left(\text{t}\right)\right)/\left[1-\left({\text{x}}_{\text{ext}}/\text{x}\left(\text{t}\right)\right)\right]\right\} \end{array}$$

(S5) $$\begin{array}{lll} \left(\text{t}/\text{g}\right)\times \ln2 & = & \ln\left\{\left({\text{x}}_{0}/{\text{x}\left(\text{t}\right)-\text{x}}_{\text{ext}}/\text{x}\left(\text{t}\right)\right)/\left[1-\left({\text{x}}_{\text{ext}}/\text{x}\left(\text{t}\right)\right)\right]\right\} \end{array}$$

(S6) $$\text{g}=(\text{t}\times \ln2)/\ln\{({\text{x}}_{0}-{\text{x}}_{\text{ext}})/\text{x}(\text{t})\times [\text{x}(\text{t})/(\text{x}(\text{t})-{\text{x}}_{\text{ext}})]\}$$

Hence:

(S7) $$\begin{array}{l} \text{g}=\left(\text{t}\times \ln2\right)/\ln\left\{\left({\text{x}}_{0}-{\text{x}}_{\text{ext}}\right)/\left(\text{x}\left(\text{t}\right)-{\text{x}}_{\text{ext}}\right)\right\} \end{array}$$

where x_0_ = 0.00366 and, in the case of *E. coli*, x_ext_ is the isotope fraction in the medium obtained from adding 9/10 volumes of 98% ^15^N (as estimated by the supplier) to 1/10 volumes of natural nitrogen:

0.9 × 0.98 = 0.882
plus 0.1 × 0.00366 = 0.000366
so x_ext_ effectively equals 0.882

Hence in this *E. coli* experiment:

(S8) $$\begin{array}{l} \text{g}=\left(\text{t}\times \ln2\right)/\ln\left\{\left(0.00366-0.882\right)/\left(\text{x}\left(\text{t}\right)-0.882\right)\right\} \end{array}$$

As an example, the population after 4 min labeling contains a cell with an isotope rate x(4) = 0.0436 which therefore has a mass doubling time of (4 × ln2)/ln{(0.00366–0.882)/(0.0436–0.882)} = 59.6 min

To validate investigations of growth rate using isotope-labeling and SIMS, we confirmed that the isotope fractions (Supplementary Figure 10) and mass doubling times (Supplementary Table 1) estimated for an individual bacterium did not vary significantly in serial sputtered sections. We then plotted the size of each bacterium against ^15^N incorporation (Supplementary Figure 11), but found no evidence that cells deviated significantly from exponential growth.

It is important to note that two individual bacteria can have different growth rates but that these growth rates are still exponential–in other words, the exponents are different (e.g., the mass of bacterium A is proportional to 2^(t/gA)^ whilst the mass of bacterium B is proportional to 2^(t/gB)^ where gA and gB are the generation times of the bacteria A and B, respectively). This exponential growth means that as bacterium A gets bigger it grows faster and that as bacterium B gets bigger it also grows faster but that, at a particular moment, bacterium A could be growing faster than bacterium B even if B were bigger than A at that same moment.

### Significance of intercellular diversity

Differences in the distribution of isotope fractions between cells are commonly used to determine whether these cells have significantly different mass doubling times. However, such differences would not be significant if they could be generated by a random distribution of isotope fractions. We therefore used simulations to determine whether the differences of isotope fractions between bacteria after a particular labeling period were significant. We did this in two ways. Firstly, we obtained the median value of all the isotope fractions of all the pixels of all the images of bacteria after a particular labeling period. We then used Maple 9.5 to simulate the isotope fractions in a population of bacteria that would be obtained by SIMS (Supplementary Figure 12). We defined a square of 240 × 240 pixels to correspond to a real SIMS image of 256 × 256 pixels. We filled this square completely with 192 bacteria each measuring 10 × 30 pixels corresponding to the region of interest (ROI) of a bacterium of 400 × 1,200 nm (in which each pixel is 40 × 40 nm). We supposed that the isotope fraction ^12^C^15^N/(^12^C^14^N+^12^C^15^N) is the same for all the bacteria and corresponds to the fraction really measured for a particular labeling period. For each pixel, we then chose as a reference the numbers of counts of ^12^C^14^N and of ^12^C^15^N that would give both this isotope fraction and correspond to the average of the counts per pixel of ^12^C^14^N and ^12^C^15^N actually measured. To represent the statistics of secondary ion counting in SIMS (Fleming and Bekken, 1995; Nikolov et al., 1996), these reference pixel values of ^12^C^14^N and ^12^C^15^N were then considered as means that were used in Maple to generate random values following a Poisson distribution. These random values then replaced the reference values in all pixels and the isotope fractions calculated. We simulated 5 images containing a total of 288,000 pixels corresponding to 960 bacteria. We calculated the median of the 288,000 pixels. For each bacterium, we then calculated the percentage of its pixels with a value below this median; we made a histogram from the numbers of bacteria (converted into a percentage) that have a particular percentage of their pixels below this median of the population and compared this histogram with one obtained from the experimentally measured values. We found in this simulation that, in a population of 192 cells with 300 pixels per cell, all cells had between 42 and 59% of their pixels below the population median.

We also repeated this simulation in a simpler way using Visual Basic 6 to obtain a random distribution of pixels either above or below the median of all the pixels in all the bacteria. This was done by taking all the pixels (equals the number of bacteria multiplied by 600 pixels per bacterium) and setting them to be blue or yellow at random whilst making the total number of blue and yellow pixels the same. These pixels were then allocated to bacteria, 600 pixels at a time, and the distribution per bacterium was analyzed. The result was similar: in a population of 200,000 bacteria, each containing 600 pixels there were no bacteria that had more than 353 pixels below the median (that is, 100% of the bacteria had between 247 and 353 blue pixels, i.e., between 42 and 59%) as shown in Supplementary Figure 13 (compare this with the experimental results shown in Supplementary Figure 14 where only 37% of the bacteria have between 42 and 59% blue pixels). Moreover, the standard deviation obtained in this simulation, 12%, was the same as that obtained for the experimental control of unlabeled bacteria.

### Index of intracellular asymmetry

To calculate the index, the pixels constituting the ROI of each bacterium were divided into two equal or nearly equal parts, P1 and P2, by a line perpendicular to the long axis such that P1 contains a total of N(P1) pixels and P2 a total of N(P2) pixels [ideally N(P1) = N(P2)]. To do this automatically, we wrote a program that first determined the rectangle that inscribed the bacterium and that then divided that rectangle into two halves (Supplementary Figure 15). If the longer side of the rectangle was an odd number of pixels, the pixels on the line bisecting this side were suppressed to prevent them from being counted twice. The median of the isotope fractions of the pixels in the cell was taken for each cell and the pixels above or below the median colored yellow or cyan, respectively. The asymmetry index, I_a_, was then calculated as the absolute value of the difference between the fraction of the cyan pixels in P1, N_C_(P1)/N(P1), and P2, N_C_(P2)/N(P2):

(4) $$\begin{array}{l} I_{\text{a}}=\text{abs}\left({\text{N}}_{\text{C}}\left(\text{P}1\right)/\text{N}\left(\text{P}1\right)-{\text{N}}_{\text{C}}\left(\text{P}2\right)/\text{N}\left(\text{P}2\right)\right) \end{array}$$

I_a_ is therefore positive and lies between 0, corresponding to no asymmetry, and 1, corresponding to a complete asymmetry. I_a_ is only weakly dependent on small differences between N(P1) and N(P2); in the ensemble of our experiments the mean of the differences between N(P1) and N(P2) was 9% which would give a deviation from the I_a_ obtained from the case of N(P1) = N(P2) of the same order of magnitude.

### Statistical analysis of intracellular asymmetry

To determine the significance of apparent intracellular asymmetries we modeled the probability of obtaining by chance a particular asymmetrical distribution of the cyan (or yellow) pixels between the two parts P1 and P2 (see above, Index of intracellular asymmetry). To simplify, we consider here that the image of the bacterium contains 2N pixels and that the parts P1 and P2 each contain N pixels. To further simplify the calculation, we assume that N is even and that N/2 is therefore an integer. Consider then a set of 2N objects (“pixels”), N being yellow and the other N, cyan. N “pixels” are drawn from the set of 2N “pixels” without replacement and are placed at arbitrary positions in the P1 part of the bacterium image. If, among the N “pixels”, a total of ((N/2)+n) cyan “pixels” are drawn from the 2N set and placed in P1, the remaining ((N/2)–n) cyan “pixels” are automatically placed in P2. The asymmetry index is therefore I_a_(n) = 2n/N with n = 0,…, N/2. Hence the probability p[I_a_(n)] of observing this index by chance is equal to the probability to draw (N/2+n) plus the probability to draw (N/2−n) cyan “pixels” (both have the same value of I_a_). These probabilities are given by a hypergeometric distribution in which the C(Q, r) is the classical binomial coefficient defined by Q!/[(Q-r)! r!] for positive integers Q and r and with Q ≥ r:

$$\begin{array}{l} \text{C}\left(2\text{N},\text{N}\right)\times \text{p}\left[{\text{I}}_{\text{a}}\left(\text{n}\right)\right]=2\times \text{C}\left(\text{N},\left(\text{N}/2\right)+\text{n}\right)\times \text{C}\left(\text{N},\left(\text{N}/2\right)-\text{n}\right) \end{array}$$

for *n* = 1,…, N/2 and C(2N, N) × p[I_a_(0)] = 2 × C(N, N/2) × C(N, N/2) for *n* = 0 (there is only a single draw corresponding to a null asymmetry index). We verified that: $\overset{N/2}{\underset{n=0}{\sum }}p\left[Ia\left(n\right)\right]=1$ as it should.

Thus, the proportion of bacteria (with 2N pixels) that have an asymmetry index lower than or equal to 2n/N is approximately cP[Ia(n)] (Supplementary Figure 16) with:

$$\begin{array}{l} cP\left[Ia\left(n\right)\right]=\overset{j=n}{\underset{j=0}{\sum }}p\left[Ia\left(j\right)\right] \end{array}$$

For each bacterium, we tested the null hypothesis in which the proportion of cyan pixels in P1 is the same as the proportion of cyan pixels in P2 (i.e., no asymmetry) and the alternative hypothesis in which the proportion of cyan pixels in P1 is not the same as in P2 (i.e., asymmetry). We used the R software to carry out an exact Fisher test based on the hypergeometric law so as to compare two proportions. We obtained a *p*-value per bacterium and made a histogram for each labeling time (Supplementary Figure 17).

Finally, we compared our index of intracellular asymmetry with the normalized difference between the average isotope ratios in each half of the cell. The two measures are very similar but the median-based index has the advantage that it is clear to the reader why we argue that the asymmetries are significant (Supplementary Figure 18).

### Choosing cells for intracellular asymmetry analysis

Two cells with different mass doubling times that come together by chance might be mistaken for a single, asymmetric cell. This possibility can be eliminated because such cells are usually at an angle to one another; where there was a doubt, we excluded the cell. Secondly, and importantly, any such “doublet” cells would be bigger than most cells but we found no evidence for an increase in intracellular asymmetry with cell size (Supplementary Figure 19).

### Estimation of diversity

A general, quantitative measure of population diversity would facilitate comparisons within and between different species of bacteria and, indeed, of eukaryotic cells; it would facilitate investigation of the effects on heterogeneity of a wide variety of growth conditions and treatments. Ideally, such a yardstick would be easy to obtain and to interpret and would be free of evident artifacts. To determine the feasibility of obtaining a measure of diversity based on isotope incorporation, we excluded labeling bacteria for short periods because estimations of the growth rates revealed significant variations from the actual growth rate (that may reflect a combination of bacterial sensitivity to slight perturbations, residual growth during chilling, variations in the samples, in detection by the NanoSIMS 50 and in the nature of growth) that would be difficult to avoid (Supplementary Figures 7, 8).

We therefore explored the possibility of using bacteria labeled for a long period. We obtained the median value of the isotope fraction [^12^C^15^N/(^12^C^14^N+^12^C^15^N)] for all of the pixels in a population of *E. coli* labeled for 128 minutes (Supplementary Figure 14A); each bacterium therefore has a percentage, p, of its own pixels above this median and (100-p) below it. A histogram was then used to give an idea of the diversity of incorporation (Supplementary Figure 14B). Since the form of the histogram depends on the arbitrary choice of the bin, we used kernel density estimation (Supplementary Figure 14C); this entails replacing each sample point of the data with a Gaussian-shaped kernel, and then summing these kernels (via Matlab). This gave distributions that could readily be compared with one another.

### Growth rate diversity and loss of material

The growth rate diversity we observe is unlikely to result from different losses of small molecules by different bacteria during sample preparation for several reasons. Firstly, glutamate, the principal intracellular source of nitrogen in *E. coli*, is at a concentration of 96 mM (Bennett et al., 2009). Differential losses of the small pool during sample preparation should therefore have released detectable quantities of glutamate into the supernatants. However, we found no evidence for the release of glutamate in any of the preparative steps (Supplementary Figure 20).

Secondly, the pool of small molecules is too low a proportion of the bacterial mass for losses in this pool to account for the CVs and mean values we observe. Metabolites, cofactors and ions constitute <4% of the dry weight of *E. coli* (and this includes molecules and ions that do not contain nitrogen; Neidhardt, 1996). Moreover, the half-lives of the small molecules containing nitrogen is very short before they are incorporated into macromolecules. Since macromolecules are made—and only made—out of small precursor molecules, it could be argued that the pool of these precursors should be incorporated into macromolecules in <4% of the generation time: consider that M.(104/100).2^T/g^ = M.2^(T+t)/g^ where M is the mass of the bacterium at time zero, T is the time after zero for which the bacterium has been growing, and t is the time needed to increase the bacterial mass by 4%; this equation gives t = 3.6 min. Hence, if it were true that the short labeling times had large CVs due to the loss of these molecules, the highest CV should be for 2 min labeling and all the subsequent CVs (e.g., for 4 and 8 min) should be much smaller. To quantify this, we wrote a program in Visual Basic 6 to simulate the effect of the random loss of the pool to different degrees in different cells. We set the program's variables for the isotope concentrations to the values corresponding to our growth rate calculations. We then used a random distribution of losses of the pool that gave the maximum CV we observed experimentally (20% after labeling for 2 min); exactly the same distribution of losses of the pools was subsequently used for the other labeling times. For a couple of 100 cells that initially had a pool of small molecules of 3%, the program gave CVs of 20, 14, 7.8, 3.9, 1.9, 0.94, and 0.47% for 2, 4, 8, 16, 32, 64, and 128 min labeling, respectively (Supplementary Table 2); these values are significantly different from the corresponding experimental values of 18, 20, 17, 12, 8, 6, and 5%. Moreover, the program shows that the random losses of the pool necessarily increase the apparent mass doubling times (losses of the pool cannot decrease them); in the case of the values obtained above by the program, the mass doubling time was estimated to be as much as 78 min, which is significantly more than the 64 min given by both our optical density and experimental SIMS measurements.

**Figure 2:**
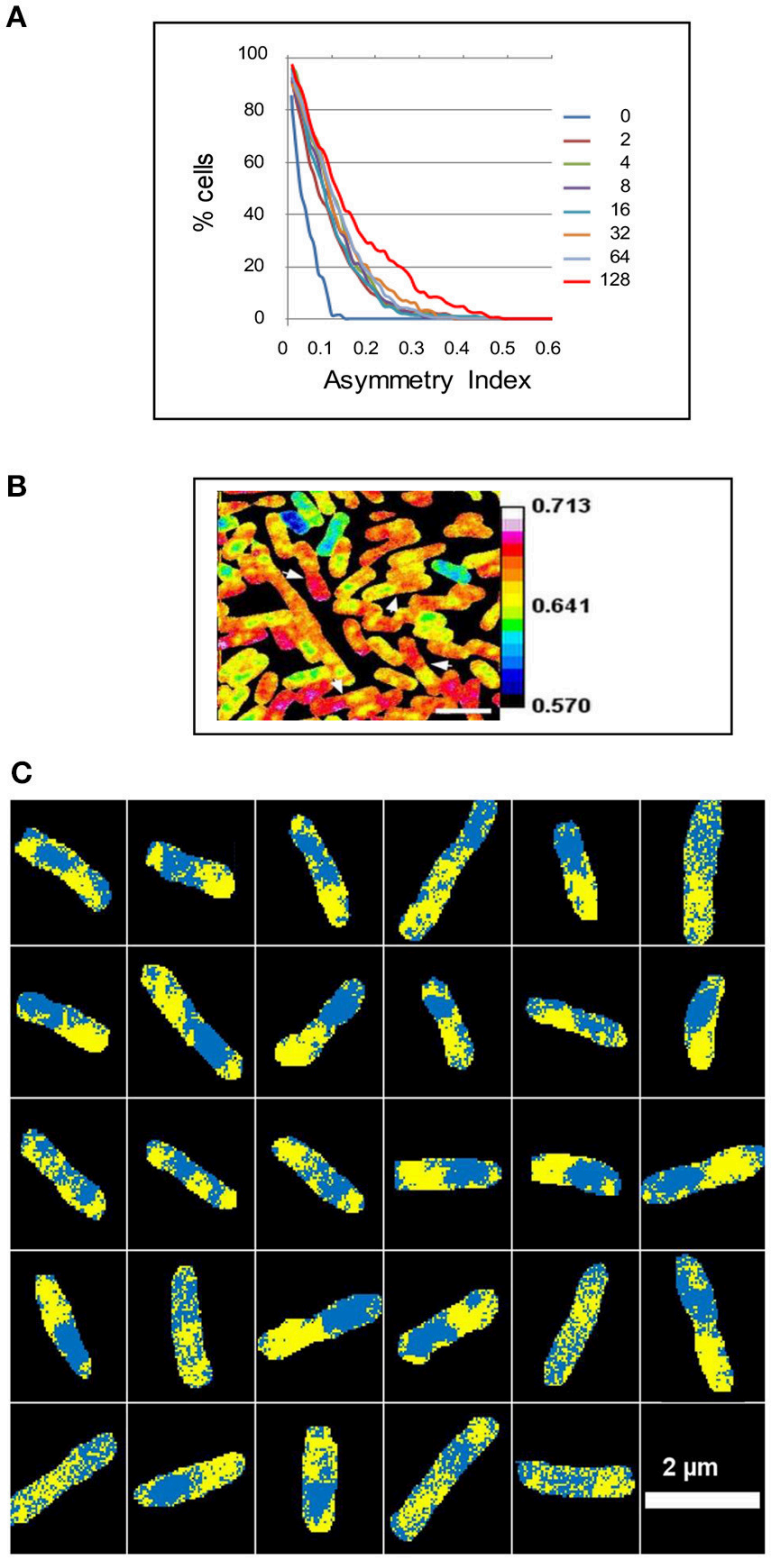
Intracellular asymmetry of isotope distribution in *E. coli*. A steady state culture was labeled with ^15^N for 2 to 128 min and analyzed using a NanoSIMS 50 to show the ratio of ^12^C^15^N/(^12^C^14^N+^12^C^15^N). **(A)** The cumulative percentage of cells 100*cP[I_a_] above a given asymmetry index I_a_ in the A series. The median was taken for each cell and the asymmetry index then calculated as the proportion of pixels above the median in one half of the cell minus the proportion above the median in the other half of the cell (see Materials and Methods). **(B)** The white arrowheads show some daughter cells that have an asymmetric distribution of the isotope after labeling for 128 min. **(C)** The median was taken for each cell after labeling for 128 min and pixels above or below the median were colored yellow or cyan, respectively, to show 29 asymmetric cells within a sample of 111 cells. Scale bar 2 μm.

Thirdly, there is no evidence of loss of molecules from specific regions of the cell. Serial sputtered sections of the same bacterium gave essentially the same isotope fractions (and hence the same estimates of the corresponding mass doubling times), consistent with any intracellular diversity resulting from losses having little effect on intercellular diversity (Supplementary Figure 10 and Supplementary Table 1). In this context, suppose that the distribution of small molecules within growing cells is homogeneous and that the loss of these molecules from regions within some of the cells during preparation is responsible for the high intercellular diversity at the short labeling periods; this loss of homogeneity within the cells should cause a high intracellular asymmetry, particularly after short labeling periods when the ratio of labeled small molecules to labeled macromolecules is highest. The opposite, however, is observed (Figure 2A).

Fourthly, differences in the binding of the ^15^N-ammonium ion to the membrane—and the loss of such ions—are unlikely to contribute significantly to the diversity since varying the regions of interest (ROIs) analyzed to include or exclude the membrane made little difference to the estimated mass doubling times (data not shown). Comparison of three successive sputtered sections of the *E. coli* cells after a short period of labeling showed that the high variation of ^15^N incorporation observed in these periods is not due to small regions of high or low incorporation that may be fortuitously present in the section analyzed. In addition, population growth rates estimated from the averages of isotope counts corresponded to those measured by optical density (Supplementary Figures 6, 8) consistent with a relatively small effect of isotope losses, particularly for the longer labeling periods.

Finally, it might be contended that adding ^15^N perturbs bacteria and thereby generates or simply exacerbates diversity. This is formally possible given that adding ^15^N can result in changes to the proteome and metabolome of *E. coli* but these changes are only minor (Filiou et al., 2012) and growing *E. coli* in a 1:1 ratio of ^15^N:^14^N is reported to result in <1% difference in growth rates (Xie and Zubarev, 2015).

### Contribution of asymmetric segregation to the estimation of growth rates

Estimation of the growth rates of the individual bacteria in the population based on isotope ratios is subject to a possible artifact, namely that an asymmetric segregation of labeled and unlabeled material into daughter cells would result in them having different isotope ratios even if their actual growth rates were the same. Hence, it might be argued that the intercellular diversity we observe is due not only to different metabolic rates but also to an asymmetric segregation of labeled and unlabeled material (Chai et al., 2014; Gupta et al., 2014). Suppose, for example, all the cells in our experiments grew with the same growth rate, then, after one generation in the ^15^N medium, up to half the cellular material would be unlabeled and, in the limit, if all this ^14^N material were concentrated in one of the two daughter cells (or, after two generations, in one of the four granddaughter cells), it would appear that this cell had not grown at all. In support of this reasoning, much of the cellular material exists in the form of large *hyperstructures* with different turnover characteristics (Norris et al., 2007; Pelletier et al., 2012; Saier, 2013), which, if they had different isotope fractions and segregated separately into the future daughter cells, might generate significant asymmetry, particularly if they affected diffusion (Parry et al., 2014).

To estimate the contribution of asymmetrical segregation to growth rate diversity, we performed three analyses: (1) we calculated the proportion of cells in the population that would have been born during the labeling period, (2) we plotted the relationship between the diversity of incorporation (which is the basis for estimating growth rates) and cell size, and (3) we calculated the growth rates that would correspond to a subpopulation of half-size cells (where each half of a cell is considered a future daughter cell); each cell therefore has a “faster-growing” half and a “slower-growing half”; these half-size cells were then classed into two subpopulations of “faster growing” or “slower growing” half-size cells; we then compared the “growth rates” of these half-size cells with the estimated growth rates of the cells themselves.

1. The total number of cells in the population after the labeling time, t, is:
  $$\begin{array}{l} {\text{N}}_{0}2^{\text{t}/\text{g}} \end{array}$$
  where N_0_ is the number of cells at the start of the labeling time and g is the mass doubling time. Noting that the number of labeled “newborn” cells is twice the increase in the number of cells over the labeling time, the number of cells born during the labeling time is:
  $$\begin{array}{l} 2\left({\text{N}}_{0}2^{\text{t}/\text{g}}-{\text{N}}_{0}\right) \end{array}$$
  Hence the proportion of labeled cells resulting from a division during labeling is:
  $$\frac{2({\text{N}}_{0}2^{\text{t/g}}-{\text{N}}_{0})}{{\text{N}}_{0}2^{\text{t/g}}}$$
  or
  $$\frac{2(2^{\text{t/g}}-1)}{2^{\text{t/g}}}$$
  This shows that the proportion of cells that result from a division during short labeling times is small being, for example, only 4% after 2 min (Supplementary Table 3).
2. Newborn cells are small so if the apparent intercellular diversity in growth rates were mainly due to asymmetric segregation of material, this diversity should only be characteristic of small cells at the short labeling times. This is clearly not the case (Supplementary Figure 11).
3. By imagining two populations of half-size cells that would be formed by the parts P1 and P2 (see above) if each cell were to divide, the isotope ratios in P1 and P2 were used to estimate their “mass doubling times” (see below) and then, for each real cell, to plot these doubling times against the estimated doubling time of the cell itself (Supplementary Figure 21); the fact that the differences within cells are much smaller than the differences between cells means that intracellular segregation can only make a small contribution to our estimates of growth rate heterogeneity even in the worst scenario where the entire population comprises cells that have divided during the labeling period, which cannot be the case for short periods (Supplementary Table 3).

## Results

### Controls based on the differentiating bacterium, *B. subtilis*

As a positive control for our investigation of phenotypic diversity in the form of metabolic diversity, an overnight culture of *B. subtilis*, a bacterium known to differentiate readily (Kerravala et al., 1964; Schaeffer et al., 1965; Veening et al., 2008), was diluted into fresh medium and grown again, as typically done in studies of its cell cycle. Once in exponential phase, ^15^N-ammonium sulfate and ^13^C-glucose were added to the culture. Cells were then collected after around two (90 min) and three (120 min) generations, and analyzed by SIMS (Supplementary Figures 4, 5). This revealed an intercellular diversity in the distribution of ^15^N and ^13^C (Supplementary Figure 4), with the distribution of ^13^C mirroring that of ^15^N (compare Supplementary Figures 2C,D). Moreover, depth-profiling the labeled cells showed that a particular pattern of incorporation extended throughout an individual bacterium (Supplementary Figure 5) and excluded the possibility that intercellular diversity upon long exposure to the label might be due entirely to a combination of intracellular heterogeneity and position of the section of the analysis.

### Using isotope fractions to estimate the mass doubling time of an *E. coli* population

We first estimated the mass doubling time from the *global* isotope fraction, namely, the values of all the pixels in all the bacteria analyzed after a particular period of labeling with ^15^N (Supplementary Figures 6–8). It should be noted that each point in these three figures corresponds to the isotope fraction of all the bacteria in a single image; note that the average of the isotope fractions of the individual images is not the same as the real global isotope fraction since the number of individual bacteria—and their corresponding isotope fractions—in an image varies. That said, these results do suggest a considerable variation in the mass doubling times estimated for the shorter labeling periods (Supplementary Figures 7, 8). One explanation is that the high sensitivity of the combination of isotope-labeling and SIMS reveals a bacterial response to a mechanical or other perturbation that occurred despite our best efforts (and that may missed by other techniques). The results of short labeling periods must therefore be interpreted with caution. However, for labeling periods over 32 min, the mass doubling time converged on 65 min which is close to the 64 min measured by the independent method of optical density. This gives us confidence that the isotope fraction is a valid measure of growth rate diversity within a population.

### Growth rate diversity and individual *E. coli* cells

Many aspects of metabolism are captured by the growth rate. This rate can be expressed as the mass doubling time, which is sometimes estimated using the isotope fractions of elements such as nitrogen as detected by SIMS following the labeling of cells (Musat et al., 2012). SIMS is a very precise technique for measuring isotope fractions particularly when the isotopes are abundant in the sample and have a high useful yield (ratio of isotopes detected to those present in the volume analyzed), as is the case for carbon and nitrogen in many biological samples (Lechene et al., 2006) (section Materials and Methods). This means that the isotope fraction ^15^N/(^15^N+^14^N) can be used to estimate with precision the mass doubling time of individual cells (section Materials and Methods); we therefore labeled cultures of *E. coli* with ^15^N for periods varying from 2 to 128 min. This revealed that the isotope fraction characteristic of each cell varied significantly in every image analyzed (Figures 1, 2). The intercellular diversity in labeling was then estimated in terms of the mass doubling times of individual cells; these times varied from 34 min to 123 min in the 4-min-labeled sample and from 65 min to 102 min in the 128-min-labeled sample (Supplementary Figure 2). The statistical significance of the intercellular diversity was confirmed using a simulation program (Supplementary Figure 12).

### Size distributions

The growth of populations of *E. coli* with different mass doubling times is associated with major changes in cell volume (Sloan and Urban, 1976; Kepes and Kepes, 1985; Ehrenberg et al., 2013). To investigate whether the differences in the mass doubling times of the individual cells in our study are related to differences in cell sizes, we estimated the sizes of these cells and obtained size distributions for the 10% of the fastest growing cells and the 10% of the slowest growing cells and compared them with the size distribution of the whole population. These distributions are similar (Supplementary Figure 22). This means that the growth of *E. coli* cells with different mass doubling times, that we report here, were *not* accompanied by major changes in cell volume. At first sight, then, our results may seem at variance with those that form the cornerstone of microbial physiology. However, the above, classical studies are based on correlating the sizes of cells with the average mass doubling times of populations growing in different media whilst our results can be likened to snapshots of the mass doubling times of individual cells within a population growing in a single medium. To put it differently, at the level of populations growing in different media, the size of the cell is correlated with the mass doubling time conferred on the population by the growth medium whereas at the level of cells *within the same population*, the size of the cell is not correlated with its mass doubling time. One possible explanation is that the mechanism responsible for the global variation in mass doubling times *between* populations growing in different media differs fundamentally from the mechanism responsible for the variation of these times *within* a single population growing in the same medium. If so, what is the nature of the mechanism responsible for generating a diversity of mass doubling times for the cells *within* a single population?

### Segregation and correct estimation of growth rates

It might be thought that if asymmetric segregation of material were indeed an important mechanism for generating different growth rates (as we propose) such asymmetric segregation would be important enough to give rise to false estimates of these growth rates. In fact, only a small proportion of cells actually divide during the short labeling times (where the growth rate diversity is greatest); moreover, the unequal distribution of label on division does not affect the *calculation* of mass doubling times significantly because the differences in label between the daughters is small. The argument that we develop below is that though this difference in distribution is sufficient to underpin the hypothesis that asymmetric segregation is responsible for generating different growth rates, it is too slight in itself to affect the estimates of growth rate.

In principle, both differences in growth rates and differences in the segregation of labeled or unlabeled material into daughter cells could contribute to the intercellular diversity in ^15^N distributions. Labeling for periods shorter than the mass doubling time of the population might be expected to give a snapshot of differences in growth rates. Labeling for periods longer than the mass doubling time (which should decrease the growth-rate-dependent diversity of ^15^N distributions) might nevertheless show persistence of an artifactual, growth-rate-*independent*, intercellular diversity if the asymmetric segregation of labeled and unlabeled material followed by cell division were an important factor in the *estimation* of growth rates.

To try to evaluate the relative contributions of growth rate and segregation-division to intercellular ^15^N distributions, we therefore compared the distributions of mass doubling times estimated after different periods of labeling (Supplementary Figure 2); the coefficients of variation (CV% = 100 × standard deviation/mean) at 2, 4, 8, 16, 32, 64, and 128 min were 18, 20, 17, 12, 8, 6, and 5%, respectively. The higher coefficients at the shorter periods are consistent with an important role for growth rate in the intercellular diversity of ^15^N incorporation whilst the fact that the coefficients remain relatively high at the longer periods is consistent with a possible role for the asymmetric segregation of labeled and unlabeled material. To investigate further the contribution of this type of asymmetric segregation to our estimates of growth rates, we reasoned that it could only affect our calculation of the growth rate of those cells that originated from a division occurring during the labeling period (which would have inherited different amounts of the label). The proportion of cells that might be affected in this way is small for brief labeling periods, being only 4% of the population labeled for 2 min (Supplementary Table 3), whereas the proportion of cells that actually differ from one another is much greater; moreover, if asymmetric segregation were responsible, only newborn cells—which are short—should be affected for brief labeling periods but there is no evidence that the subpopulation of short cells has a greater intercellular diversity than that of long cells (Supplementary Figure 11). To estimate the *maximum* contribution that intracellular asymmetry of labeled and unlabeled material might make to the estimation of intercellular growth rates, the pixels constituting the image of each bacterium were divided into two equal or nearly equal parts (Supplementary Figure 15) and the ‘growth rates’ corresponding to each part were estimated separately. Plotting the estimated growth rate of each of these imaginary, half-size cells against the estimated growth rate of the cell itself shows that the variation in incorporation within cells is too small to generate the observed variation in incorporation between cells from which growth rates are estimated (Supplementary Figure 21). Taken together, these results mean that the asymmetric segregation of labeled and unlabeled material can only have a small effect on the actual estimation of growth rates, particularly after periods of labeling much briefer than the mass doubling time.

### Growth rate diversity and the cell cycle

**Figure 3:**
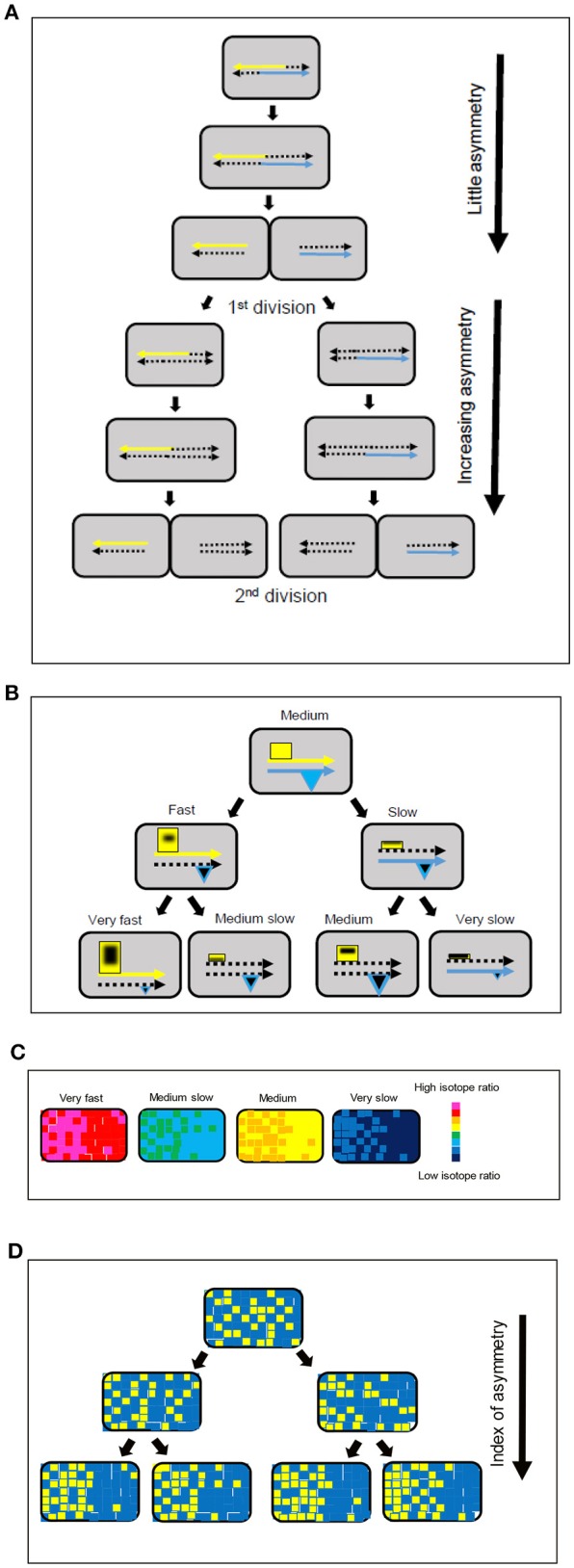
Model for strand-directed diversity and asymmetry. **(A)** The labeling of the chromosome and associated macromolecules during replication and segregation makes a greater contribution to intracellular asymmetry after the first division. **(B)** Positive feedback and association with the parental DNA strands leads to an increase in the size of hyperstructures (yellow rectangles) responsible for fast growth in one daughter cell and a decrease in the size of these hyperstructures in the other daughter cell, as shown in the left-hand branch; this tendency continues into the next generation. There is a corresponding decrease in expression of the hyperstructures responsible for fast growth, as shown in the right-hand branch; in parallel, expression of the hyperstructures responsible for survival (blue triangles) can either be maintained or decrease. The continuous yellow and blue arrows represent the parental DNA strands, the dotted arrows represent newly synthesized strands, and the black shapes within the hyperstructures represent ^15^N-labeled material. **(C)** Growth rate diversity in the four, second generation cells in the bottom line of **(B)** shown using a chromatic scale. **(D)** Increase in index of intracellular asymmetry (arrow) shown by classing pixels above (yellow) or below (blue) the median of the isotope ratio for the pixels within a single bacterium for the four, second generation, cells in **(B)**. Comparison of the same cells in **(C,D)** shows that there is no contradiction between the evidence for the lack of importance of the intracellular asymmetry of labeled/unlabeled material in the actual estimation of mass doubling times **(C)** and the evidence for the importance of strand-based asymmetry as a mechanism for generating intercellular diversity **(D)**.

What are the origins of the extensive growth rate diversity we and others observe (Godin et al., 2010; Campos et al., 2014; Taheri-Araghi et al., 2015; Wallden et al., 2016)? One possibility is that it originates in the cell cycle, which is intimately linked to growth rate diversity in differentiating bacteria such as *Caulobacter crescentus*. We have previously proposed that the cell cycle in both differentiating and non-differentiating bacteria generates a coherent metabolic diversity because the parental DNA strands and physically associated macromolecules are segregated during the replication of the chromosome such that subsequent division results in one daughter cell receiving macromolecules responsible for slow growth and the other daughter cell receiving macromolecules responsible for fast growth (Rocha et al., 2003). These sets of macromolecules are part of the class of molecular assemblies termed *hyperstructures* that perform specific functions at intracellular locations defined in part by their association with the chromosome (Norris et al., 2007; Llopis et al., 2010). It might therefore be expected that these hyperstructures would have different rates of turnover according to their function, composition and location. We reasoned that these different rates of turnover might, in principle, translate into differences in our labeling experiments in the intracellular distribution of ^15^N so, to explore the possible involvement of the cell cycle in the generation of growth rate diversity, we obtained the median value of the pixels of each individual cell and displayed pixels above or below that value in yellow or cyan, respectively; we then divided each cell roughly into two halves (corresponding to the future daughter cells) and estimated asymmetry as an index, I_a_, calculated as the absolute value of the fraction of cyan pixels in one half minus the fraction of cyan pixels in the other half. To determine the significance of these estimates, we obtained the probability distributions of the asymmetry index (Supplementary Figure 17). Except for the control, most of the *p*-values are small and the null hypothesis of no significant asymmetry can be rejected. We then plotted the cumulative percentage of bacteria as a function of the asymmetry index after each labeling period. An increase in asymmetry was striking after 128 min labeling (Figure 2). To quantify the significance of this asymmetry, we used the hypergeometric distribution of yellow and cyan pixels within a cell. This showed that the probability of observing by chance a bacterium with an asymmetry index >0.16 is <0.001 (Supplementary Figure 16). This probability distribution corresponds to that obtained for the unlabeled control bacteria (Figure 2A and Supplementary Figure 16). For the labeled bacteria, however, all curves show many bacteria with asymmetry indices >0.16 which is highly unlikely to occur by chance (Supplementary Figure 16); for example, 15% of the bacteria after 16 min labeling have an asymmetry index >0.16 although the probability of observing by chance a single bacterium with an asymmetry index >0.16 is <0.001, whilst at 128 min, over 30% of the bacteria had an index over 0.16 (Figure 2A). Why does asymmetry increase up to 128 min? 128 min corresponds to the time that would be needed for cells growing with the same rate of 64 min to undergo two mass doublings. In the classic experiment of Meselson and Stahl (Meselson and Stahl, 1958), an increase in asymmetry of labeled material from one to two mass doublings revealed the semi-conservative replication of DNA. Our results may therefore be explained if the labeled and unlabeled DNA strands plus associated hyperstructures (themselves containing different proportions of labeled and unlabeled material) were to follow the pattern of DNA distribution characteristic of semi-conservative replication and hence if the cell cycle itself via the distribution of these different hyperstructures were indeed to be a general and major determinant of growth rate diversity (Figure 3).

### Toward a standard measure of growth rate diversity

The distribution of growth rates of the individuals in a population is an important characteristic of a species because it reflects the overall capacity of the species to respond to its environment. This environment can include, of course, the presence of antibiotics. There are several ways that the isotope fraction, [^12^C^15^N/(^12^C^14^N+^12^C^15^N)], might be used to measure growth rate diversity. Ideally, such measurements should be standardized and, for example, samples might be taken at times based on the mass doubling time of the population as here where samples were taken at 2, 4, 8, 16, 32, 64, and 128 min, that is, 2^x^ (where −5 ≤ x ≤ 1) of the doubling time of 64 min as measured by optical density. The shorter periods of labeling show the sensitivity of isotope incorporation to physical and/or chemical stimuli, which, though interesting in its own right, means that such periods are unsuitable for estimating doubling times in steady state (section Materials and Methods). For the A series, the estimated doubling times after 2, 4, 8, and 16 min labeling periods are clearly too different from the steady state doubling time (as measured by optical density) to be useful whereas, for the B series, only the 2 and 4 min periods are different. The distribution of growth rates is one measure of diversity (see above) whilst the distribution of the asymmetry indices, I_a_, is another. The distribution of growth rates can be usefully expressed as the distribution of mass doubling times (Supplementary Figure 2); to facilitate comparisons between different species growing in different conditions with very different average growth rates, we propose to represent the diversity of estimated growth rates using the growth rate index of the single cell, I_g_, defined as:

(the difference between the estimated mass doubling time of the bacterium and the mass doubling time of the sample population)/the mass doubling time of the sample population

where the mass doubling time of the sample population is estimated using the mean isotope fraction of all the bacteria in the sample after a particular labeling period. Hence, I_g_ = 0, I_g_ > 0 and I_g_ < 0 for a bacterium growing with a mass doubling time that is the same as, greater than, or less than of the population, respectively. One advantage of using I_g_ is that the standard deviation of its distribution is identical to the familiar coefficient of variation of the mass doubling times. The relative growth rate measured by I_g_ is underpinned by the structuring of intracellular activity that is measured by I_a_. We therefore propose that each cell sampled after each labeling period be characterized by these two indices (Supplementary Figure 23) whilst the population at each time be characterized by the standard deviation of I_g_ (since the mean of I_g_ is zero by definition) and by the mean and standard deviation of I_a_ (Supplementary Table 4), thereby providing quantitative measures for studies of growth rate diversity.

## Discussion

### Growth rate diversity

It has been argued that the laws underlying cell growth—and, in particular, those directly relevant to the cell cycle such as growth rate—are to be found not by studying single cells but rather by studying cells as an aggregate (Cooper, 2006). This argument is weakened by evidence for growth rate diversity in exponentially growing cultures of *E. coli* cells, which video-microscopy has shown grow with different rates (Kiviet et al., 2014) that may vary two- to four-fold (Wang et al., 2010; Campos et al., 2014; Osella et al., 2014). A very different, high precision technique, which depends on trapping individual *E. coli* in a resonator and determining their buoyant densities, has also shown a wide variety of growth rates (Godin et al., 2010). To complement these approaches, we have investigated growth rate diversity using the combination of labeling with stable isotopes and analysis by SIMS, as commonly used for studies of metabolism in microbial ecology. This combination has the advantages of being quantitative and precise and of allowing isotopes to be localized on the scale of 50–100 nm; using this combination, we have obtained evidence for a considerable intercellular diversity of incorporation of isotopes for all labeling periods in a population of the non-differentiating bacterium, *E. coli*, growing in liquid medium in steady state conditions. This diversity largely reflects growth rates that can vary four-fold even in a sample of a couple of 100 cells. We then extended this study to the very different model system of *B. subtilis* where we also obtained evidence for a considerable intercellular diversity (Supplementary Figure 4).

What then is the explanation for this growth rate diversity? Clearly, this explanation may involve fluctuations in gene expression and metabolic cycles (Korobkova et al., 2004; Tu et al., 2007; Peterson et al., 2015). An alternative or additional possibility is that the cell cycle—which comprises the replication of the chromosome, the segregation of the chromosomes, and cell division—is itself responsible for generating growth rate diversity. In support of this possibility, cell division is important in establishing growth rates (Reshes et al., 2008) and in generating diversity in bacteria (Musat et al., 2008; Rego et al., 2017; Yu et al., 2017). The segregation of intracellular material in *E. coli* is characterized by asymmetry (Lindner et al., 2008; Wang et al., 2010; Chai et al., 2014; Bergmiller et al., 2017). Such segregation, followed by cell division, might also generate a coherent metabolic diversity: in the strand-specific model, which has a eukaryotic echo (Klar, 1987), the strand-specific hyperstructures that separately accompany each of the parental DNA strands are segregated to separate positions during the replication of the chromosome; this results in one of the daughter cells receiving hyperstructures that steer it toward a slow growth phenotype whilst the other daughter cell receives hyperstructures that steer it toward a fast growth phenotype (Rocha et al., 2003). It has indeed been found that genes and their products are located together (Llopis et al., 2010) and that the leading and lagging strands are in different locations (White et al., 2008). To explore further this idea, we analyzed the asymmetric distributions of ^15^N within cells after different periods of labeling. This distribution was striking after 128 min, that is, after an average of two mass doubling times (Figure 2). One seductive interpretation is that this reflects the combination of the semi-conservative nature of DNA replication (Meselson and Stahl, 1958) and the strand-specific phenotype (Rocha et al., 2003).

How exactly might the strand-based separation of hyperstructures generate coherent diversity? Our results are consistent with the operation of two related processes (Figure 3). In this hypothesis, the first process is the positive feedback nature of the distribution of transcriptional and translational resources according to the parental DNA inherited by the daughter cell (the areas of the rectangles and the triangles in Figure 3B correspond to the sizes of the different hyperstructures); this positive feedback occurs because there is competition within the cell for these resources during chromosome replication such that the hyperstructures that are physically associated with a parental strand (e.g., by coupled transcription-translation) and, in particular, the hyperstructures responsible for fast growth, outcompete their equivalents on the new strand (left branch, Figure 3B); hence there is a corresponding decrease in those “fast growth” hyperstructures that are associated with the new strand (e.g., cell labeled “medium slow,” Figure 3B). The second process is the positive feedback nature of the distribution of resources between growth strategies (which includes RNA polymerases, ribosomes and transcription-translation in general) and survival strategies (which includes DNA-binding proteins and storage materials). There are several reasons why this positive feedback might occur; for example, transcriptional activators might preferentially associate with one another and with their binding sites on one DNA strand, or the accumulation of survival-related material might reduce transcription-translation from the chromosome of one of the future daughter cells; this latter possibility is illustrated by the greater combined area of the hyperstructures in the “medium growth” cell compared with the combined area of these hyperstructures in the “very slow growth” cell at the right of the bottom line of Figure 3B. Material that is synthesized during the labeling (black shapes within the hyperstructures in Figure 3B) is distributed differently across the bacterial population to give the isotope ratios per pixel shown schematically in Figure 3C.

### Semi-conservative distribution of hyperstructures

The combination of isotope ratios and medians is extremely sensitive and, by taking the median value of the isotope ratios of all the pixels in a bacterium and displaying these pixels as yellow or blue according to whether they are above or below this median, respectively, we find that labeled and unlabeled material is distributed heterogeneously (Figure 2C). This is to be expected since much of this material is in the form of hyperstructures. This material is, however, also distributed asymmetrically; again, this might be expected since the location of certain hyperstructures will be determined by the location of corresponding regions of DNA, which are themselves located in particular places in the cell (as, for example, in *C. crescentus* Viollier et al., 2004). In analyzing this asymmetry, it is significant that the first generation of daughter cells has a relatively low asymmetry index but that the second generation of cells has a high asymmetry index (Figure 3), which echoes the evidence for semi-conservative replication (Meselson and Stahl, 1958) and which is consistent with the major role of strand separation and hyperstructures in producing diversity or, more precisely, is consistent with hyperstructures with different metabolic activities segregating with one or other of the DNA strands into the future daughter cells (Rocha et al., 2003).

The results presented here on intracellular and intercellular diversity, taken together with the results from other approaches, have implications for the control of the phenotype by the cell cycle. These results are consistent with a major role for the cell cycle in the generation of a heterogeneous but phenotypically coherent population of cells that travel between the two attractors of growth and survival and that are ready to deal with a wide diversity of challenges and opportunities. This role would entail the growth rate being reset during the cell cycle; the operation of a mechanism to do this might explain the puzzling observation that the cells in our experiments had similar diameters despite their different growth rates. *E. coli* growing with generation times shorter than the time required for replication of the chromosome should be undergoing multi-fork replication and be wider (to accommodate the extra DNA) than cells growing much more slowly (Schaechter et al., 1958; Zaritsky and Pritchard, 1973; Zaritsky, 2015). A cell growing rapidly would not need to change its diameter if its growth rate were not maintained beyond division and, consistent with this, no significant differences in diameter were reported in a microfluidics study showing widely different growth rates (Wang et al., 2010).

The results presented here also have implications for the control of the cell cycle itself. Control over cell division has recently been attributed to the combination of a “sizer” and a “timer” (Osella et al., 2014) or to a constant increase in volume between divisions (Campos et al., 2014; Taheri-Araghi et al., 2015). In the latter case, the hypothesis has been extended to the control over the initiation of DNA replication (Taheri-Araghi, 2015). Control over the cell cycle can, however, be attributed to factors that are directly related to phenotypic diversity such as (1) the composition in terms of macromolecular assemblies or hyperstructures, responsible for survival and growth, and (2) the intensity of activity of these hyperstructures (Norris and Amar, 2012). In this hypothesis, the cell cycle not only generates cells with different combinations of hyperstructures appropriate for growth or survival but also *is itself driven* by these combinations. Evidence for this role of hyperstructure dynamics in cell cycle control may come from more information about the activity of hyperstructures via isotope labeling and SIMS.

### Measures of growth rate diversity

In 1949, Jacques Monod remarked: “The study of the growth of bacterial cultures does not constitute a specialized subject or branch of research: it is the basic method of Microbiology” (Monod, 1949). The growth rate diversity of a culture might therefore be considered basic to Microbiology. Certain aspects of this diversity are captured by the distribution of isotope incorporation in a steady state population and it could also be argued that this distribution is a unique characteristic of a species. As such, the growth rate distribution could serve as a yardstick to estimate the effects of treatments that include alterations to the genome and addition of antibiotics. One question therefore is how best to represent the growth rate distribution. In addition to the usual distribution of mass doubling times and their means and standard deviations, in the case of SIMS analysis of isotope-labeled cells, we suggest that at least two other parameters should be measured: the growth rate index, I_g_, and the intracellular asymmetry index, I_a_. I_g_ can be obtained from the estimated growth rates; these estimates do not require correcting since the calculations themselves are not greatly affected by the segregation of labeled and unlabeled material. That said, our data indicate that the strand-specific segregation of hyperstructures is an important determinant of growth rate; the extent of this segregation is revealed by I_a_. Given the importance of the cell cycle in generating diversity, we also suggest that cells should be labeled for periods that are directly related to the mass doubling time.

## Author contributions

GYG: Performed experiments and analyzed data; CR, AC-F, AD, GG, GC, and VN: Analyzed data; DG, CL-B, EL, PG, LJ, and J-NA: Performed experiments. All authors helped write the paper.

### Conflict of interest statement

The authors declare that the research was conducted in the absence of any commercial or financial relationships that could be construed as a potential conflict of interest. The reviewer TDB and handling Editor declared their shared affiliation.
