# Supplementary material for: Division-Based, Growth Rate Diversity in Bacteria
Source: Front Microbiol. 2018 May 11;9:849. doi: 10.3389/fmicb.2018.00849 (PMC5958220; doi:10.3389/fmicb.2018.00849)
Supplement: Supplementary file 1 [file Image_1.pdf]

Supplementary Material

Division-based, growth rate diversity in bacteria

G. Yannick Gangwe Nana[1], Camille Ripoll[2], Armelle Cabin-Flaman[3], David Gibouin[3], Anthony Delaune[3], Laurent Janniere[4], Gerard Grancher[5], Gaelle Chagny[5], Corinne Loutelier-Bourhis[6], Esther Lentzen[7], Patrick Grysan[7], Jean-Nicolas Audinot[7] and Vic Norris[1]*

*** Correspondence:** Vic Norris: victor.norris@univ-rouen.fr

Supplementary Figure 1 | Absence of artefactual incorporation of ^{15}N during preparation of cells. To confirm that our results were not affected by a possible incorporation of isotopes during the preparation of the bacteria, *E. coli* was grown and prepared in exactly the same conditions as for the labeling experiments except that ^{14}N medium was used for growth (rather than ^{15}N) and that growth was stopped by adding M9 ^{15}N medium at 0°C (see Material and Methods). The ratios of $^{12}C^{15}N/(^{12}C^{14}N+^{12}C^{15}N)$ were obtained with a NanoSIMS 50. Scale bar 2 µm

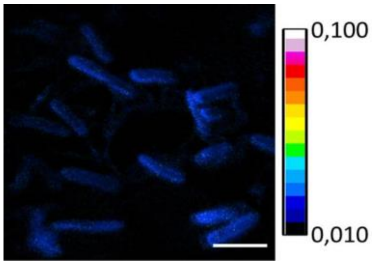

Supplementary Figure 2 | Estimated mass doubling times of individual cells differ within the same population. *E. coli* was labeled with ^{15}N in three independent experiments (**A**,**B**,**C**,**E**; **D,F**; **G**) for times of (**A**) 4 min. (**B**) 32 min. (**C**),(**D**) 64 min. (**E**),(**F**),(**G**) 128 min. Cells were fixed with ethanol (**A-F**) or formaldehyde without ethanol (**G**). Mass doubling times were estimated using equation (S7). The bins correspond to [0;36[, [36;38[, [38;40[, [40;42[... [96;98[, [98;100[, [100;...[

A

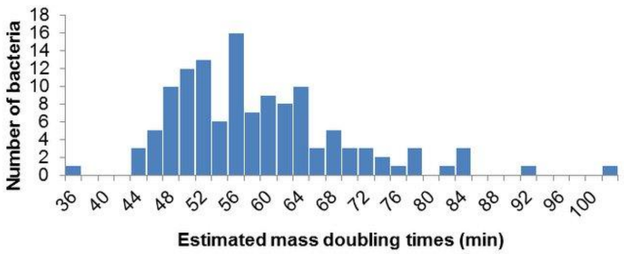

B

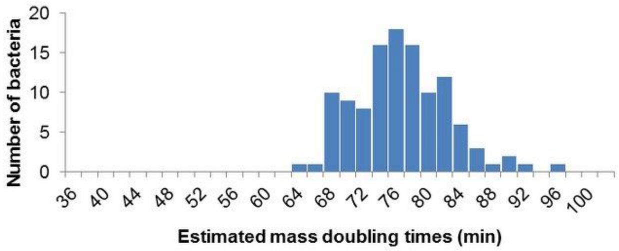

C

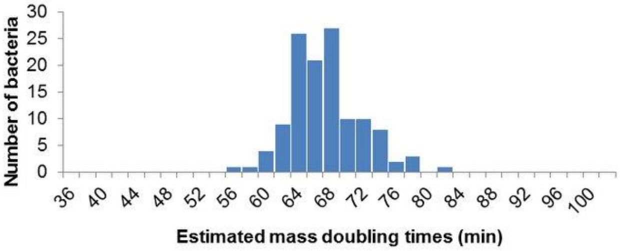

D

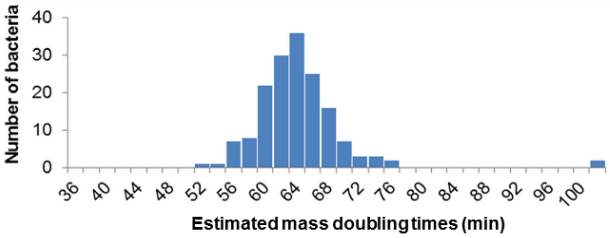

E

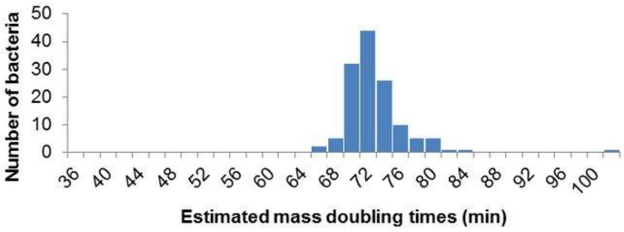
Number of bacteria
50
40
30
20
10
0
36 40 44 48 52 56 60 64 68 72 76 80 84 88 92 96 100
Estimated mass doubling times (min)

F

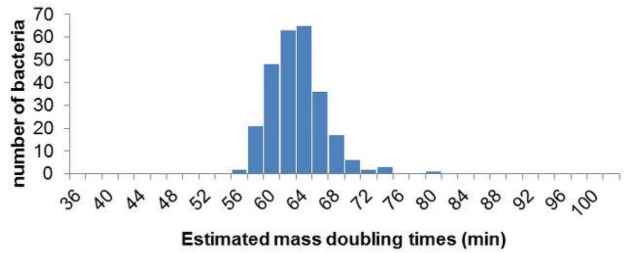
number of bacteria
70
60
50
40
30
20
10
0
36 40 44 48 52 56 60 64 68 72 76 80 84 88 92 96 100
Estimated mass doubling times (min)

G

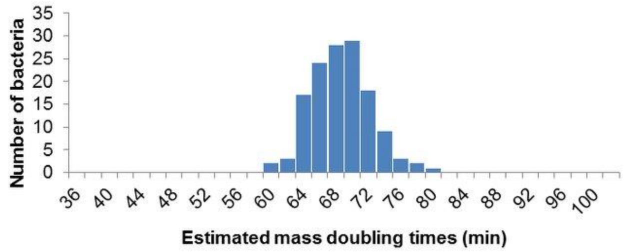
Number of bacteria
35
30
25
20
15
10
5
0
36 40 44 48 52 56 60 64 68 72 76 80 84 88 92 96 100
Estimated mass doubling times (min)

Supplementary Figure 3│Electron micrographs of *E. coli* show no evidence for lysis. The cells were prepared for analysis and, following the ethanol dehydration step, were then covered with carbon and analyzed by (A) scanning electron microscopy. (B) Secondary Ion Mass Spectroscopy to detect electrons following bombardment by the electron gun on the NanoSIMS 50 (note that the resolution is inferior to that of scanning electron microscopy. (C) Secondary Ion Mass Spectroscopy to detect $^{12}C^{14}N^{-}$ following pulverisation by the primary Cs^{+} ion beam on the NanoSIMS 50. It should be noted that though the same cells have been analyzed in (B) and (C), the cells look very different. This is because the bombardment by the electrons does not damage the cells whereas the Cs^{+} ion beam sputters them away as it generates the secondary ions (which constitute the SIMS image); moreover, the generation and detection of the secondary ions is subject to a range of artefacts (e.g. due to topography) that affect the image of a single species, e.g., $^{12}C^{14}N^{-}$ as here, but that are largely avoided by imaging the isotope ratio.

A

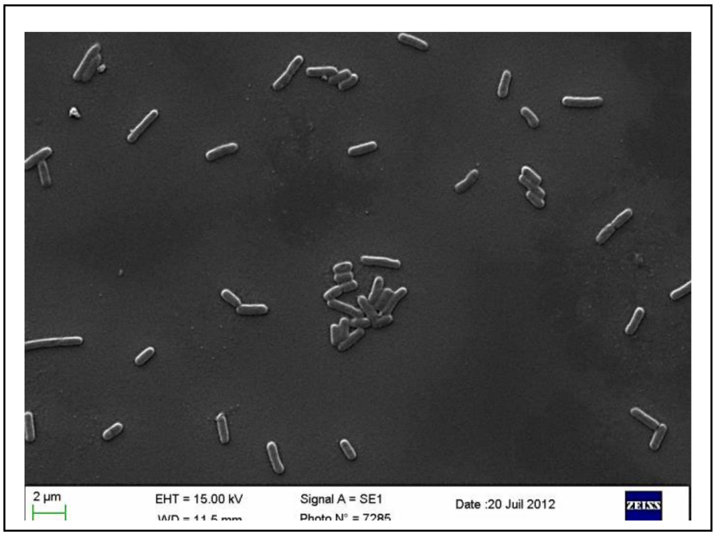

B

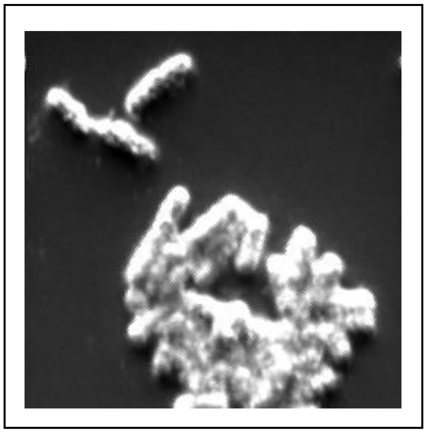

C

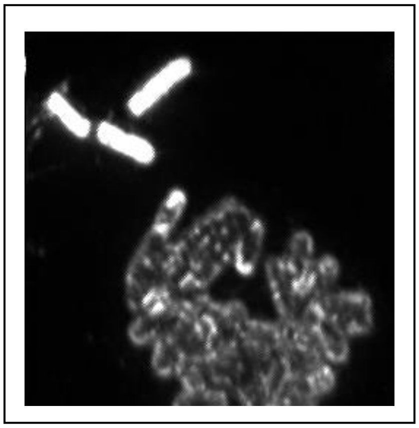

Supplementary Figure 4 │ Heterogeneous growth of *B. subtilis*. Exponentially growing cells were labeled using 50% ^{15}N-ammonium sulfate and 50% ^{13}C-glucose for 90 minutes (**A**, **B**, **C, D, E**) or 120 min (**F**) and analyzed with a NanoSIMS 50. (**A**) $^{12}C^{14}N$. (**B**) $^{12}C^{15}N$. (**C**) $^{12}C^{15}N/(^{12}C^{14}N+^{12}C^{15}N)$. The arrowheads show cells that had significantly different levels of isotope incorporation (mainly mauve or mainly blue pixels). (**D**) $^{13}C^{14}N/(^{12}C^{14}N+^{13}C^{14}N)$ with the chromatic scale adjusted to show similar distributions of nitrogen and carbon. (**E**) The $^{13}C^{15}N$ distribution. (**F**) $^{12}C^{15}N/(^{12}C^{14}N+^{12}C^{15}N)$ after labeling for 120 min. Scale bar 2 µm. The chromatic scale of the pixels corresponds to 16 equal divisions between the maximum and minimum of the range of either isotope counts (**A**, **B**, **E**) or ratios (**C**, **D**, **F**).

A

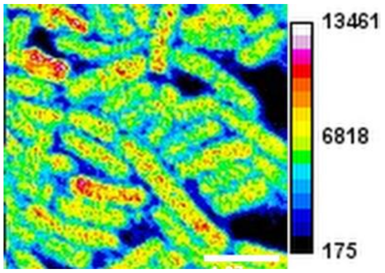

B

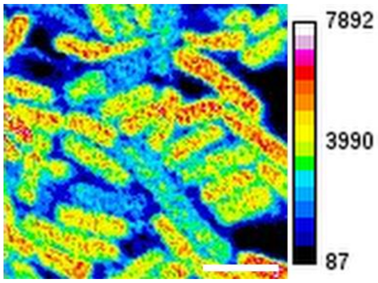

C

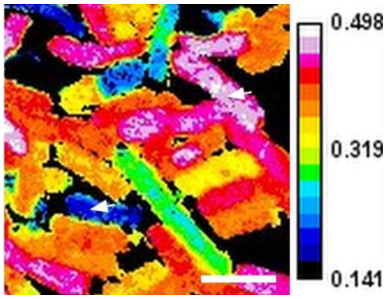

D

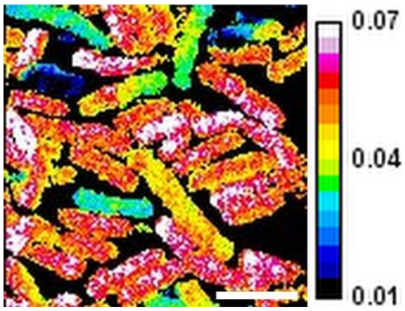

E

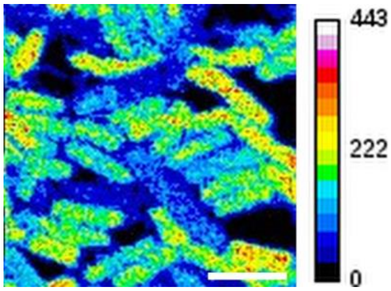

F

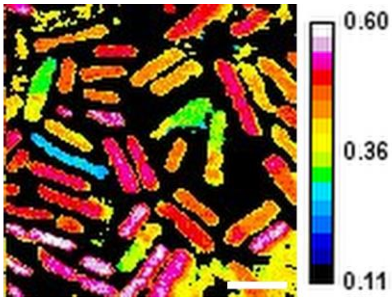

Supplementary Figure 5 | Depth profiling of *B. subtilis* labeled with ^{15}N. After labeling cells for 90 min with ^{15}N and ^{13}C, cells were analyzed with a NanoSIMS 50 to obtain the ratio of $^{12}C^{15}N/(^{12}C^{14}N+^{12}C^{15}N)$ in 12 successive sputtered sections (1 to 12, correspond to the order in which the sections were obtained). Each section here is about 7 nm thick. The ratio of $^{12}C^{15}N/(^{12}C^{14}N+^{12}C^{15}N)$ confirms that the isotope distribution shown in **Figure 1** is not limited to a single sputtered section. Scale bar 2 μm

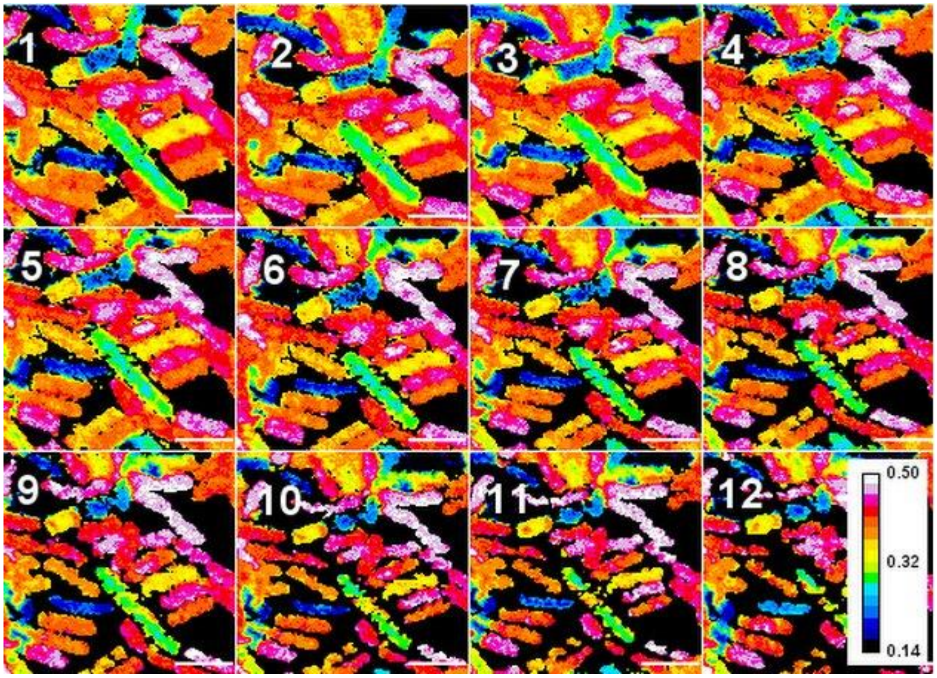

Supplementary Figure 6 | Growth of *E. coli* subpopulations measured in separate images as a function of the labeling period. Plot of the experimental values $\overline{Y_j(t)} = (x_{ext} - x_0)/(x_{ext} - \overline{x_j(t)}) = \exp(\gamma t)$ (equation (3) in Material and Methods) as a function of the labeling period, t. x_{ext} = the fraction of ^{15}N to total N in the final medium after addition of the ^{15}N, x_0 = the natural fraction of ^{15}N, t is the period of labeling, g is the mass doubling time, and $\gamma = (\ln 2)/g$. The continuous line is the least squares best fit of an exponential function a*exp(b*t) to these values. A (blue) and B (pink) are two independent series. Each point in the figure corresponds to the isotope fraction of all the bacteria in a single image; note that the average of the isotope fractions of the individual images is not the same as the real global isotope fraction since the number of individual bacteria – and their corresponding isotope fractions – in an image varies.

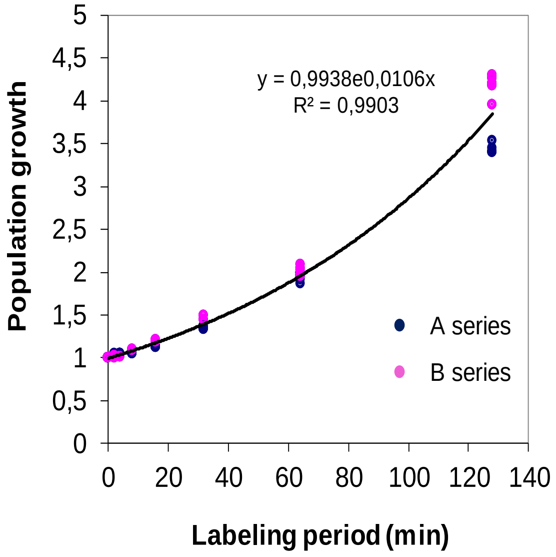

.

Supplementary Figure 7 | Variation of the isotope fraction x(t) in separate images as a function of the labeling period. The image mean values $\overline{x_j(t)}$ for two independent series, A (blue) and B (pink) of *E. coli* labeled for different periods with 90% ^{15}N (see Material and Methods) were plotted and the theoretical exponential model calculated with a doubling time of 65 min (see **Supplementary Figure 6**). Bars are the standard deviations. A better fit is observed after the longest labeling periods (64 and 128 min) than after the shorter periods (see the zoom in the right of this figure). Each point in the figure corresponds to the isotope fraction of all the bacteria in a single image.

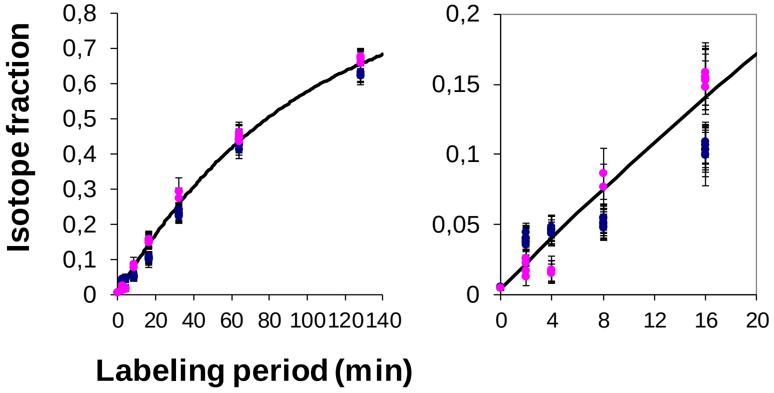

Supplementary Figure 8 | Variation of the estimated mass doubling times of subpopulations in separate images as a function of the labeling period. The deviations from the exponential growth model occurring after short periods of labeling *E. coli* with 90% ^{15}N are even clearer when the "instantaneous" mass doubling time:

$$g = t*(\ln 2)/\ln(\overline{Y_j(t)}) \quad \text{(S9)}$$

is represented as a function of the labeling period. The A series is blue and B series pink. The horizontal line is at 65 min (see **Supplementary Figure 7**). The addition of the bacteria to the medium containing the label results in a mechanical and/or chemical perturbation. The bacteria apparently respond with a transient change in growth rate which can be studied using the sensitive combination of isotope labeling and SIMS. After half a mass doubling time (32 min), the population has recovered exponential growth. The difference between the two series after short labeling periods is clear and the data from these periods must therefore be interpreted with caution.

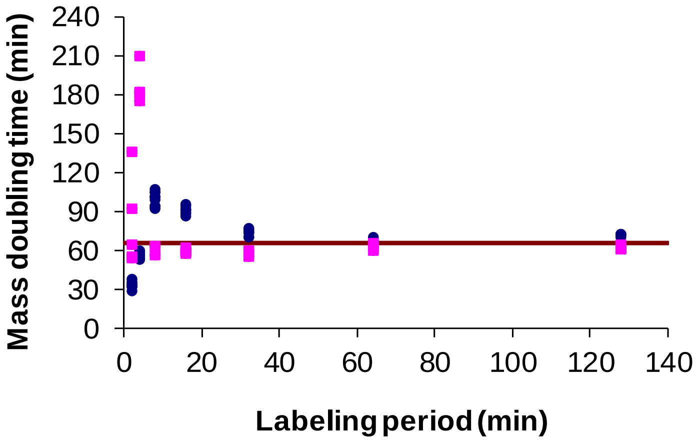

Supplementary Figure 9 | Absence of a major perturbation affecting cell division. The cell size distribution remained unchanged over the period of the experiment, indicating (1) that any perturbation due to the transfer to the medium enriched in ^{15}N did not affect cell division and (2) that the cells were growing in steady state. Average cell size, red line; bars are standard deviations.

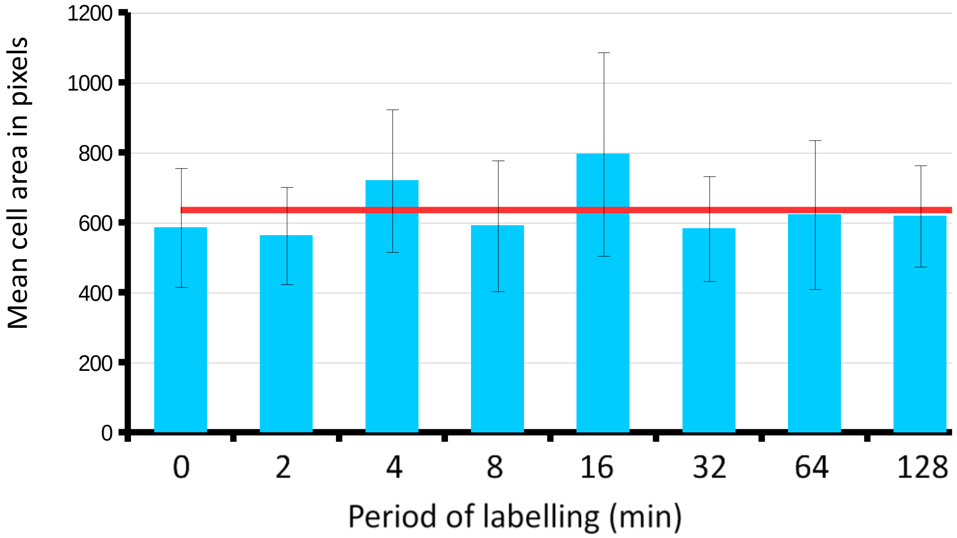

Supplementary Figure 10 | Consistency of isotope fractions obtained from sputtered sections of *B. subtilis*. Essentially the same isotope fractions were obtained from analysis of the means of all the bacteria in each of the serial sputtered sections (one mean per section) in **Supplementary Figure 5**.

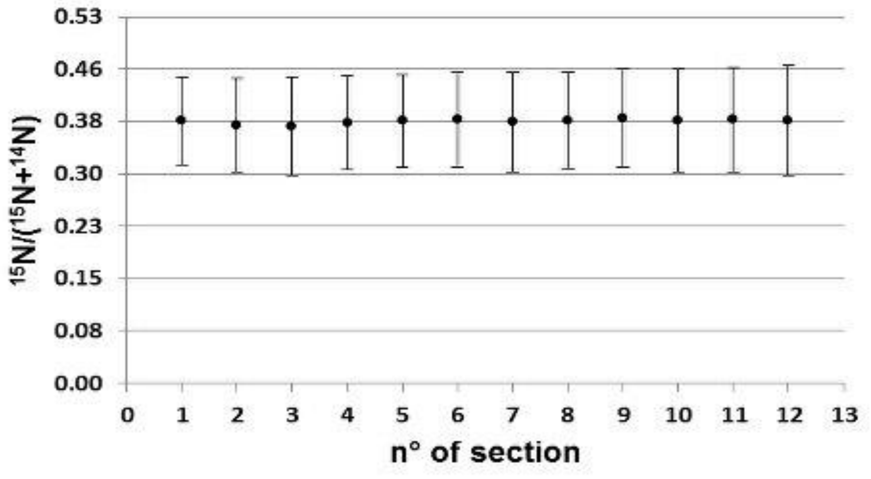

Supplementary Figure 11 | Exponential growth of *E. coli* confirmed using isotope counts and size. The number of ^{15}N atoms counted in each bacterium using a NanoSIMS 50 after a particular period of labeling with 90% ^{15}N was plotted against the size of that bacterium in pixels (note that the diameter of *E. coli* in these conditions was constant and the bacteria were lying on a flat surface so bacterial size is proportional to the area measured). The points can be fitted by a straight line consistent with incorporation per unit mass being constant and the bacteria growing exponentially over the cell cycle. Labeling for (**A**) 2 min. (**B**) 4 min. (**C**) 8 min. (**D**) 16 min. (**E**) 32 min. (**F**) 64 min. (**G**) 128 min.

A

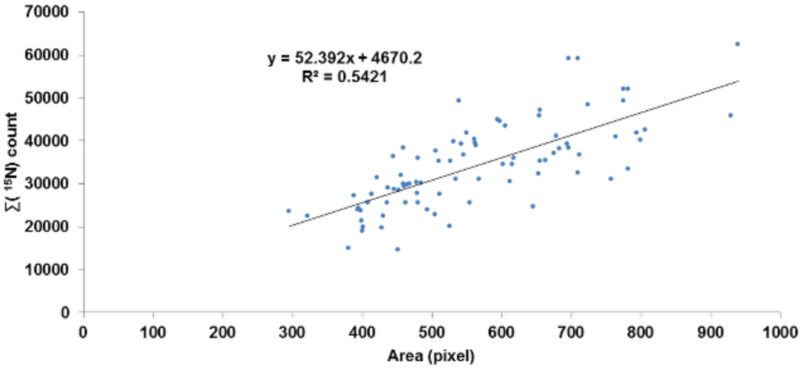

B

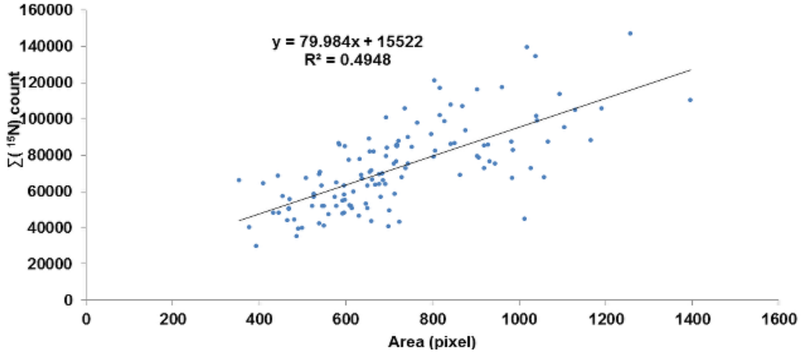

C

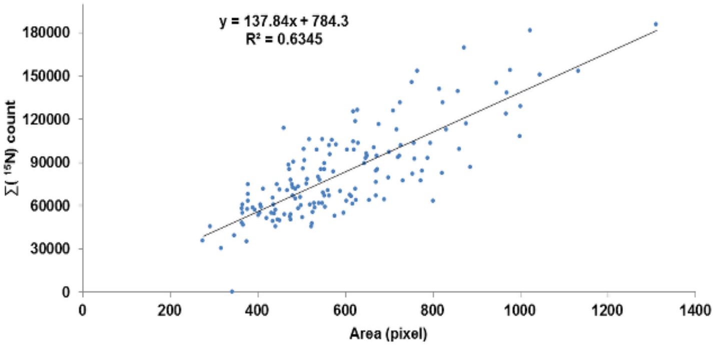

D

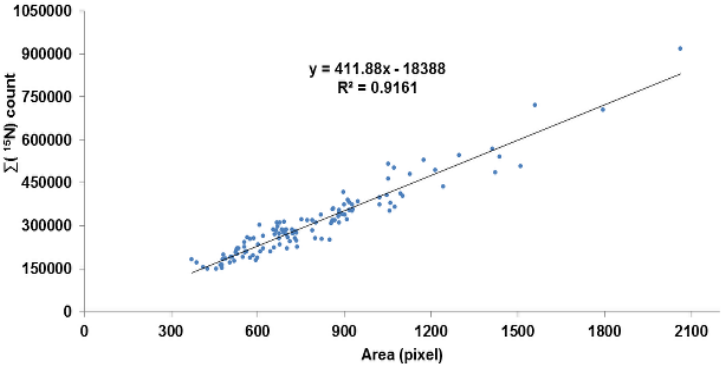
y = 411.88x - 18388
R² = 0.9161
∑(¹⁵N) count
1050000
900000
750000
600000
450000
300000
150000
0
0
300
600
900
1200
1500
1800
2100
Area (pixel)

E

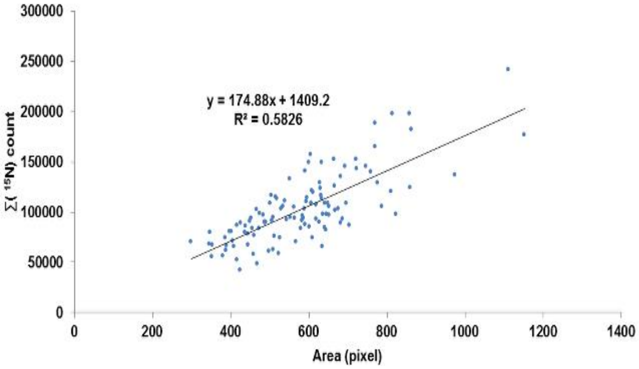
y = 174.88x + 1409.2
R² = 0.5826
∑(¹⁵N) count
300000
250000
200000
150000
100000
50000
0
0
200
400
600
800
1000
1200
1400
Area (pixel)

F

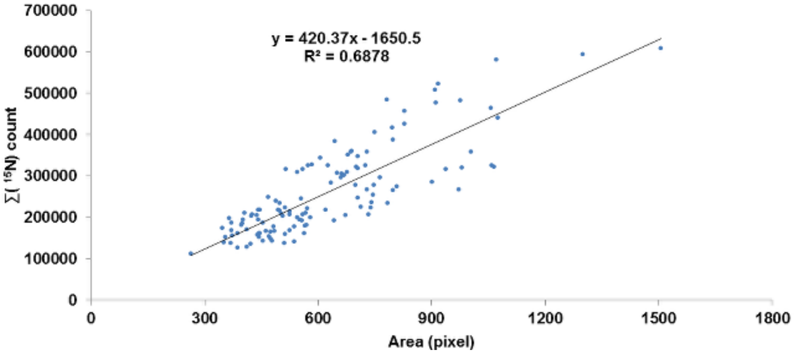
y = 420.37x - 1650.5
R² = 0.6878
∑(¹⁵N) count
700000
600000
500000
400000
300000
200000
100000
0
0
300
600
900
1200
1500
1800
Area (pixel)

G

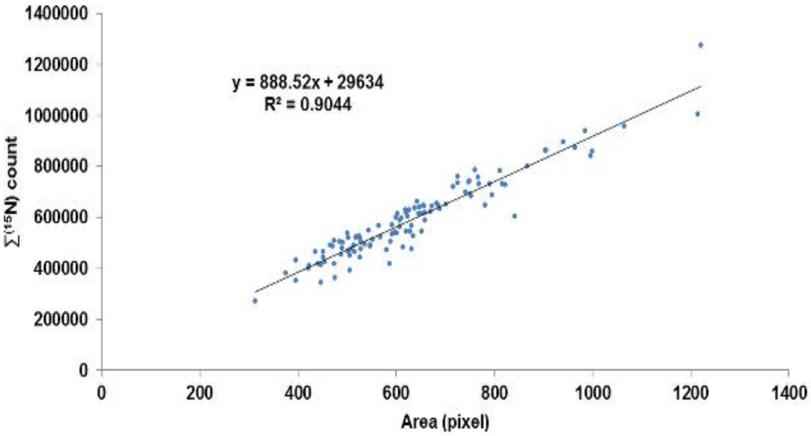
y = 888.52x + 29634
R² = 0.9044
∑(¹⁵N) count
1400000
1200000
1000000
800000
600000
400000
200000
0
0
200
400
600
800
1000
1200
1400
Area (pixel)

Supplementary Figure 12 | Isotope distribution within a population. A steady state culture of *E. coli* was labeled with ^{15}N for different periods. The median isotope fraction $^{12}C^{15}N/(^{12}C^{14}N+^{12}C^{15}N)$ was calculated for the population and then the percentage of pixels in each bacterium below this median was determined (pale blue); the bins were obtained by dividing by 25 the range of the maximum and minimum values of this percentage. A simulation program (Material and Methods) was used to generate the distribution of the percentage of cells that could be expected by chance to have a particular percentage of their pixels with isotope fractions below the median of the pixels of the population of all the cells (cyan). Typical results are shown for labeling for (**A**) 32 min. (**B**) 128 min.

A

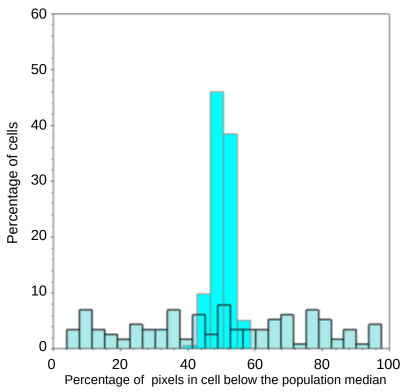

B

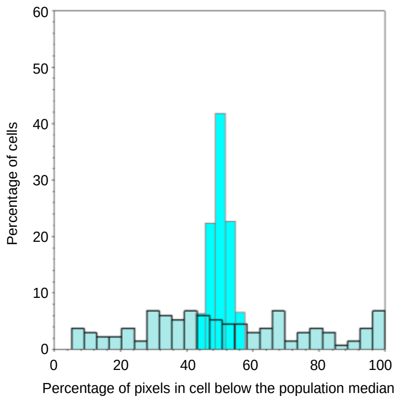

Supplementary Figure 13 | Distribution of pixels below and above the median of the bacterial population randomly attributed to cells. To investigate the significance of our experimental results on the diversity of growth rates, Visual Basic 6 was used to simulate the distribution of pixels below the median (termed 'blue') of the isotope ratios of pixels in an entire population of bacteria all growing at the same rate. There were 600 pixels in each of 200,000 cells and the bin size is 4% (as in **Supplementary Figure 12**). The median was 300-301 pixels. In this particular simulation, the standard deviation was 12, the %CV was 4, and no cell had more than 353 blue pixels or less than 247 blue pixels. In our samples of up to a few hundred cells (**Supplementary Figure 12**), there were cells in all bins and some cells were almost entirely blue or yellow (**Supplementary Figure 14**).

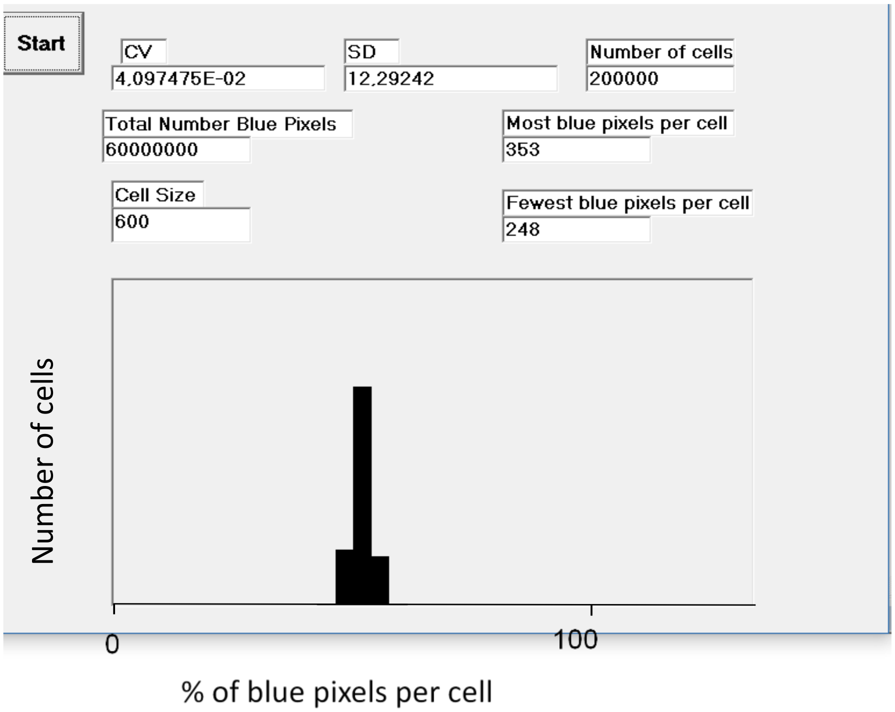

Supplementary Figure 14 | Intercellular diversity of isotope distribution in a population of *E. coli*. A steady state culture was labeled with ^{15}N and analyzed using a NanoSIMS 50 to obtain the ratio of $^{12}C^{15}N/(^{12}C^{14}N+^{12}C^{15}N)$. (**A**) The median was calculated for the isotope fraction of all pixels of the entire population of bacteria after labeling for 128 min; yellow pixels have values above this median and cyan pixels have values below it. (**B**) Histogram of percentage of pixels in the image of each bacterium below the population median after labeling for 128 min. (**C**) Kernel density estimation of the distribution of pixels in the population of bacteria after different periods of labeling.

A

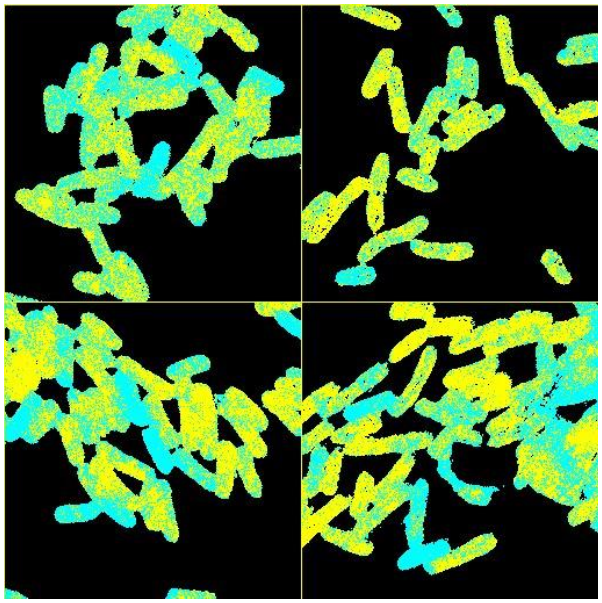

B

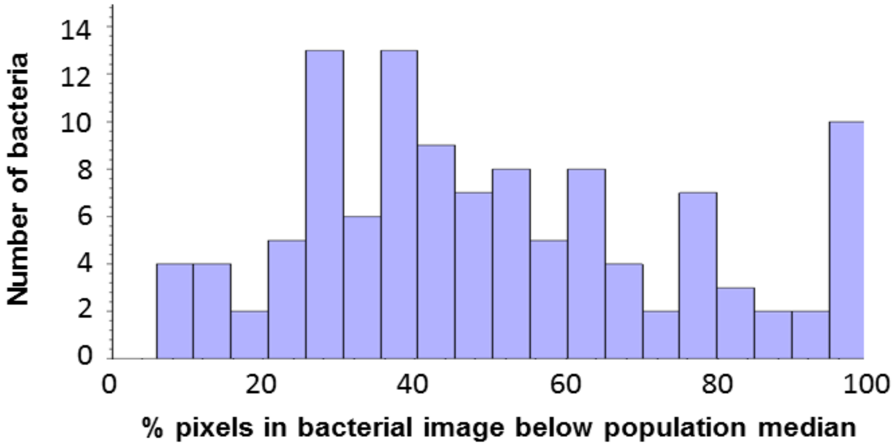

C

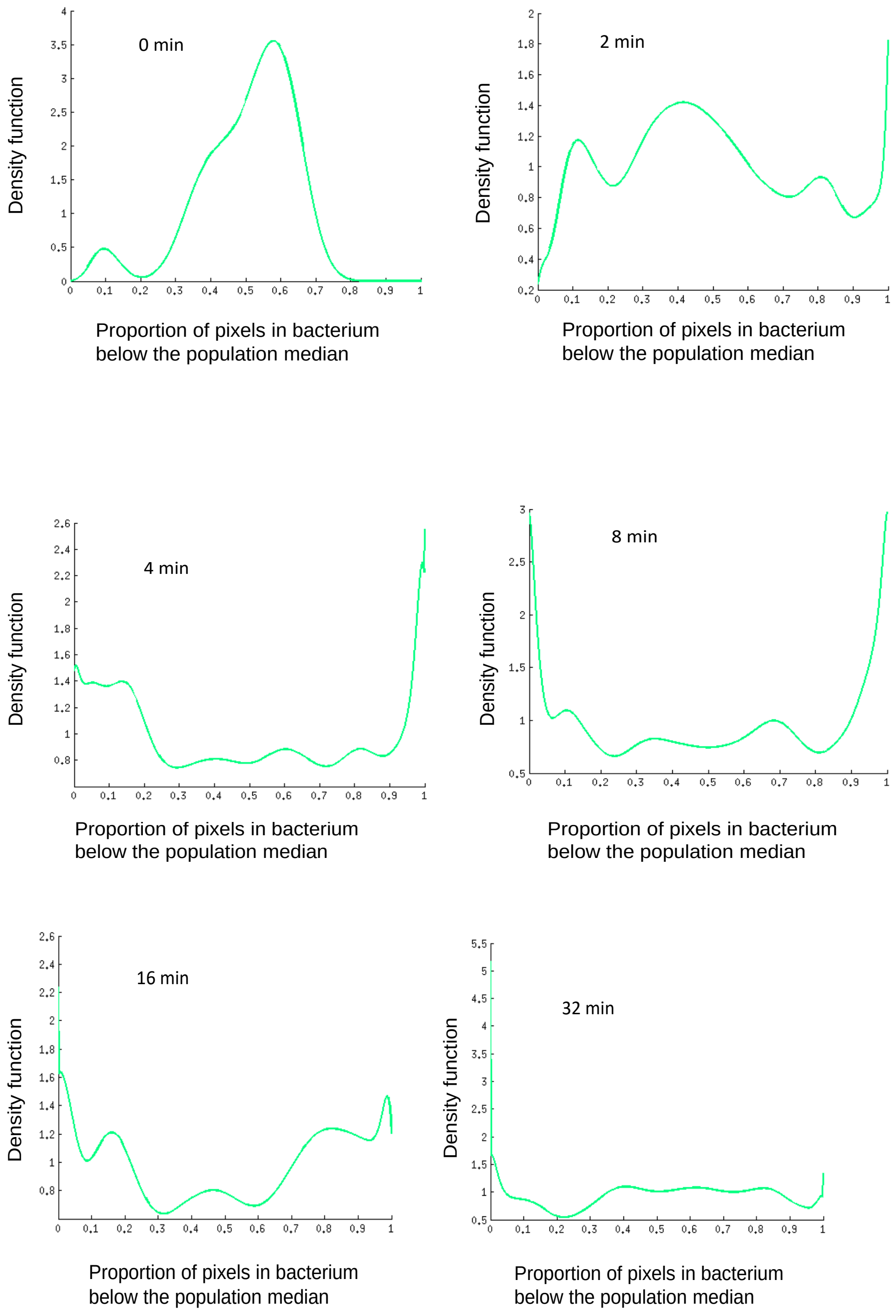
0 min
Density function
Proportion of pixels in bacterium below the population median
2 min
Density function
Proportion of pixels in bacterium below the population median
4 min
Density function
Proportion of pixels in bacterium below the population median
8 min
Density function
Proportion of pixels in bacterium below the population median
16 min
Density function
Proportion of pixels in bacterium below the population median
32 min
Density function
Proportion of pixels in bacterium below the population median

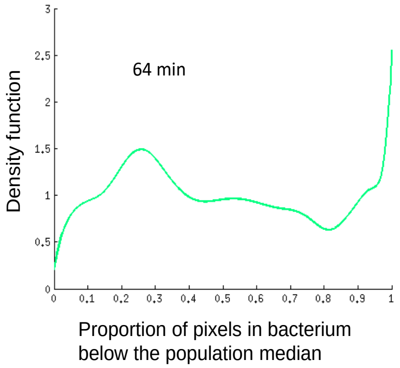
64 min
Density function
Proportion of pixels in bacterium below the population median

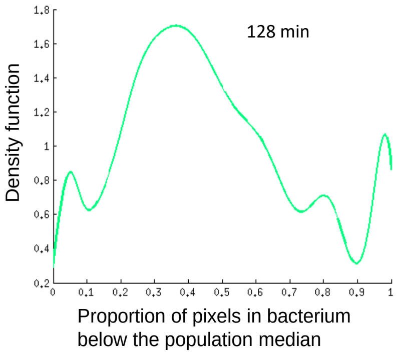
128 min
Density function
Proportion of pixels in bacterium below the population median

Supplementary Figure 15 | Division of a bacterium into two parts. The program first determined the coordinates of a rectangle that inscribed the bacterium and then divided that rectangle into two halves **(**Material and Methods**).** The median was taken for each cell and the pixels above or below the median colored yellow or cyan, respectively. The asymmetry index, I_a, was then calculated as the absolute value of the difference between the fraction of the cyan pixels in part 1 and the fraction of the cyan pixels in part 2. Part 1 and part 2 are defined for a pixel with respect to its position either above or below the dividing line (red) as here or, when the long side of the rectangle is horizontal, to the left or right of the dividing line. Hp1 = height of part 1, Hp1 = height of part 2, red line (axis) = limit between parts 1 and 2

.

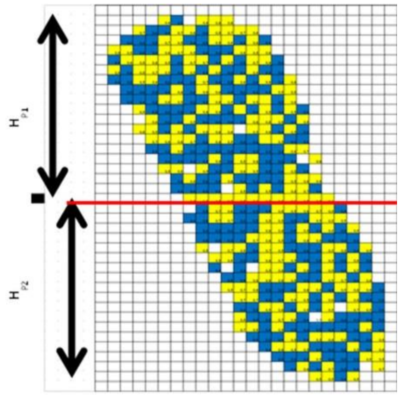

Supplementary Figure 16 | Significance of intracellular asymmetry. To evaluate the significance of the intracellular asymmetries shown in **Figure 2**, the asymmetry index I_a was estimated for a population of virtual cells of different sizes using the hypergeometric distribution (Material and Methods). The cumulative percentage of cells 100*cP[I_a] over a particular asymmetry index I_a was then plotted. For comparison, the experimental results for unlabeled control bacteria and for the bacteria labeled for 16 min labeling period (typical of the short labeling periods) are shown again (**Figure 2A**). Note that the image of the average-sized bacterium has 600 pixels.

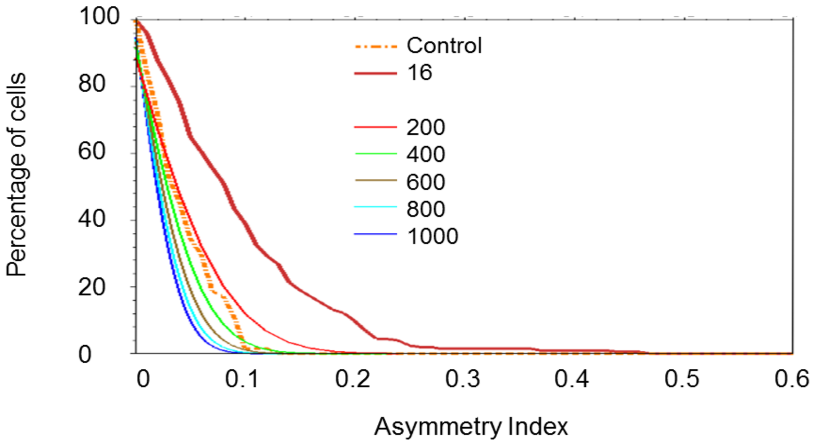

Supplementary Figure 17 | Probability distributions of the index of asymmetry. After labeling *E. coli* with 90% ^{15}N for different periods, the number of bacteria with a particular value of the asymmetry index was plotted against the probability of that value of the index by chance. In part (**A**), for example, there are 8 bacteria for which the p-value of the Fisher test is greater than 0.95 (this corresponds to bacteria that have close to an asymmetry index of 0, i.e., with very similar numbers of cyan and yellow pixels in the part analyzed); there are also 6 bacteria for which the p-value of the Fisher test is greater than 0.05 and less than 0.1; in part (**B**), more than 80 bacteria have a p-value of less than 0.05. (**A**) 0 min. (**B**) 2 min. (**C**) 4 min. (**D**) 8 min. (**E**) 16 min. (**F**) 32 min. (**G**) 64 min. (**H**) 128 min.

A

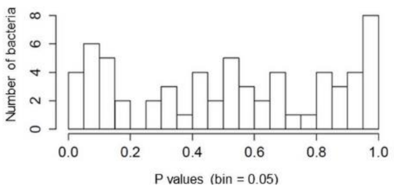

B

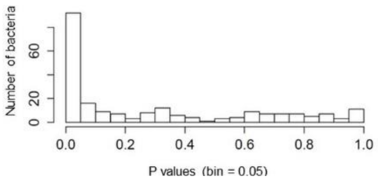

C

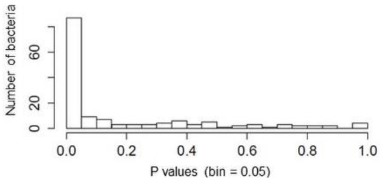

D

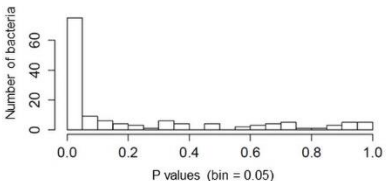

E

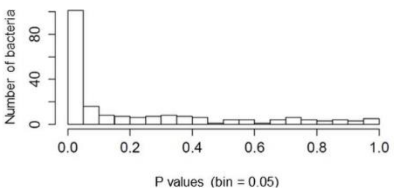

F

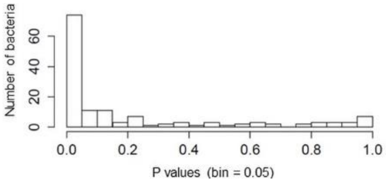
Number of bacteria
0
20
40
60
0.0
0.2
0.4
0.6
0.8
1.0
P values (bin = 0.05)

G

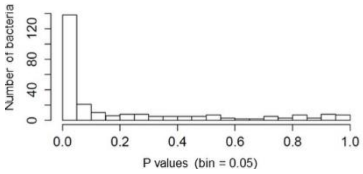
Number of bacteria
0
40
80
120
0.0
0.2
0.4
0.6
0.8
1.0
P values (bin = 0.05)

H

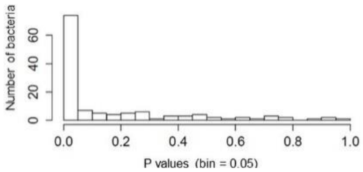
Number of bacteria
0
20
40
60
0.0
0.2
0.4
0.6
0.8
1.0
P values (bin = 0.05)

Supplementary Figure 18 | Linear relationship between measures of intracellular asymmetry. Asymmetry based on the median (the Asymmetry Index) was plotted against asymmetry measured as the normalized difference of the average isotope ratios in each half of the cell (IR asymmetry). Labelling for periods of: (A) 32 min. (B) 64 min. (C) 128 min.

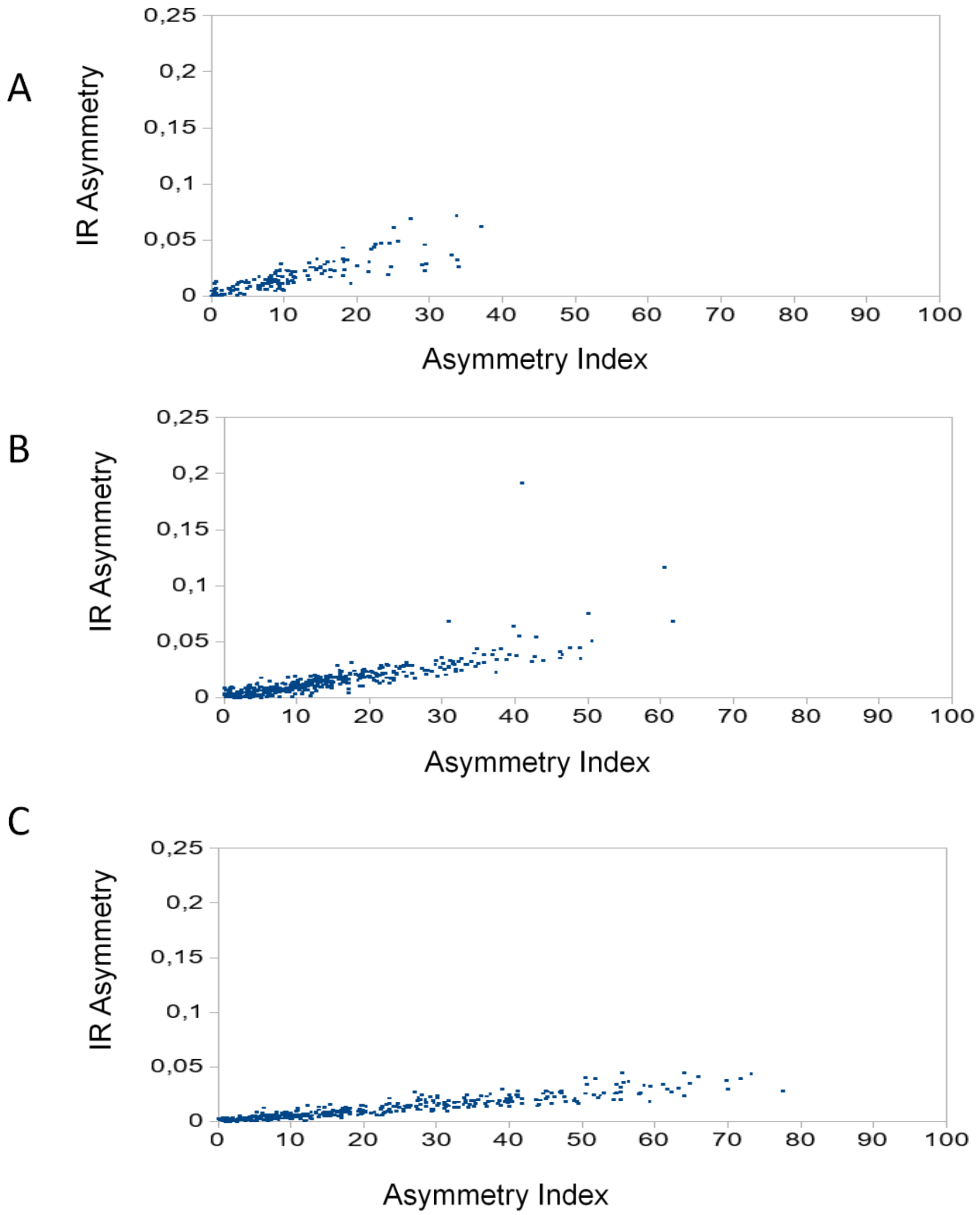

Supplementary Figure 19 | No clear relationship between intracellular asymmetry and cell size. The asymmetry index was plotted against the area in pixels of each cell. Labelling for periods of: (A) 2 min. (B) 64 min. (C) 128 min.

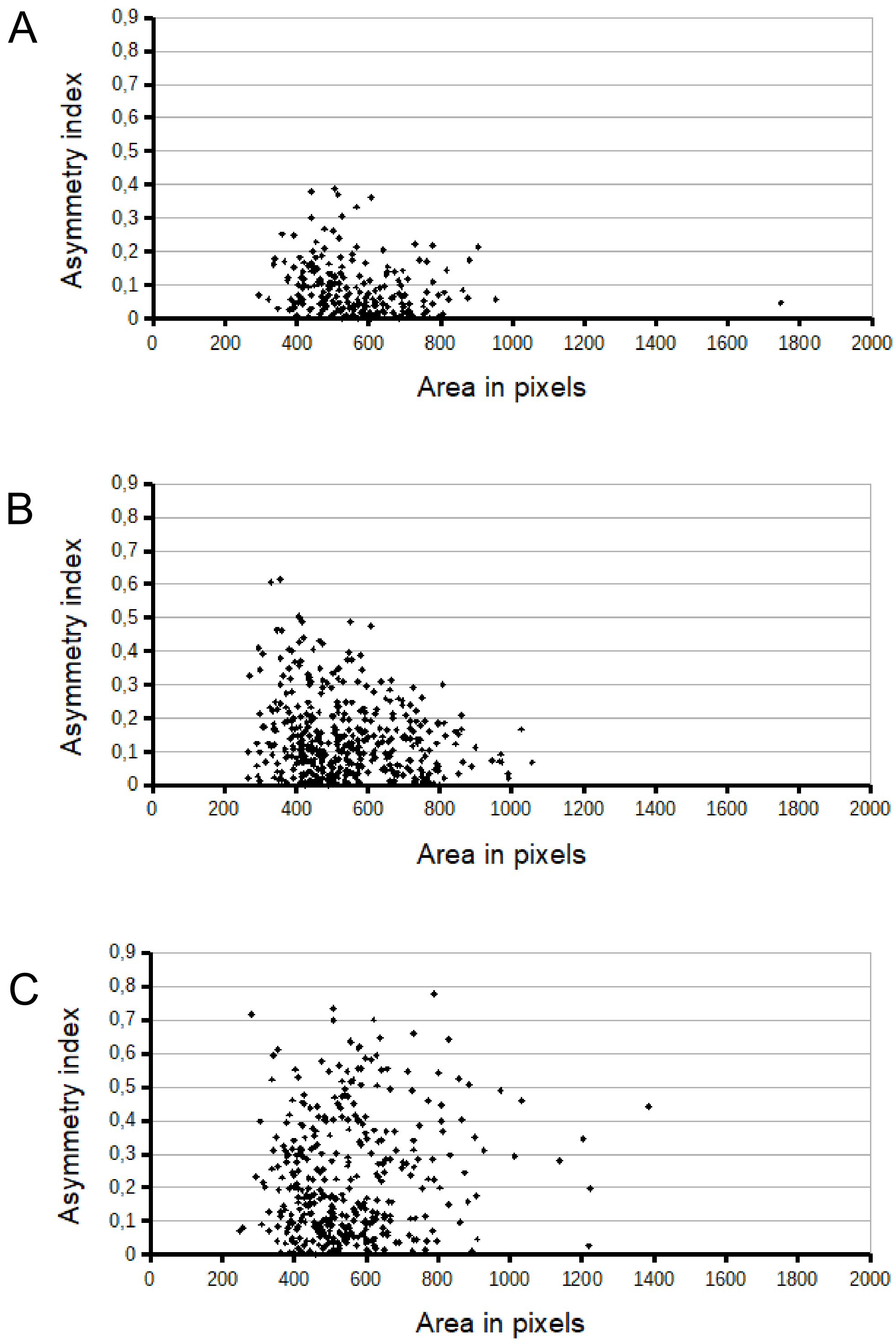

Supplementary Figure 20 | Release of glutamate from *E. coli*. To investigate the possibility that the intercellular diversity results from the loss of small, labeled or unlabeled, molecules during sample preparation, cells growing in steady state were prepared as described in Material and Methods. Supernatant fluids from all the sample preparation steps were stored on ice for the mass spectrometry analysis. Electrospray ionization mass spectra as well as *Selected Reaction Monitoring* "*m/z* 146 → *m/z* 128" experiments (insets) were acquired for all steps. Note that the ion at *m/z* 137 is cacodylate and that some of the differences in the spectra are due to normalisation to the most abundant species (100%) and to the ions in the solvent (e.g., *m/z* 195, *m/z* 175 and *m/z* 119). (**A**) positive control showing detection of glutamate at a threshold concentration of 6.8 x 10^{-8} mol/L corresponding to the lysis of about $5x10^4$ cells of average volume 1 μm^3, each containing 100 mM glutamate, in 100 µl. The deprotonated molecule of glutamate (*m/z* 146) was detected as well as the peak corresponding to the glutamate specific precursor-to-product ion transition "*m/z* 146 → *m/z* 128". (**B**) absence of detection of glutamate release in a sample of 1.28 x 10^9 cells in 100 µl fixed with formaldehyde (note that the samples analyzed by SIMS that we report here contained 10^9 cells in 100 µl); neither the deprotonated molecule of glutamate (*m/z* 146) nor the peak corresponding to the glutamate specific precursor-to-product ion transition "*m/z* 146 → *m/z* 128" were detected. (**C**) absence of detection of glutamate release following the first treatment with 25% and 50% ethanol; neither the deprotonated molecule of glutamate (*m/z* 146) nor the peak corresponding to the glutamate specific precursor-to-product ion transition "*m/z* 146 → *m/z* 128" were detected. In conclusion, none of the preparative steps showed the presence of glutamate which, if released in any step, would have been by less than about 1:10^4 cells ($5x10^4$/1.28 x 10^9) or, put differently, glutamate release would be less than 0.01% of the total pool of glutamate in the average cell, i.e., 10 µM (0.01% of 100 mM).

A

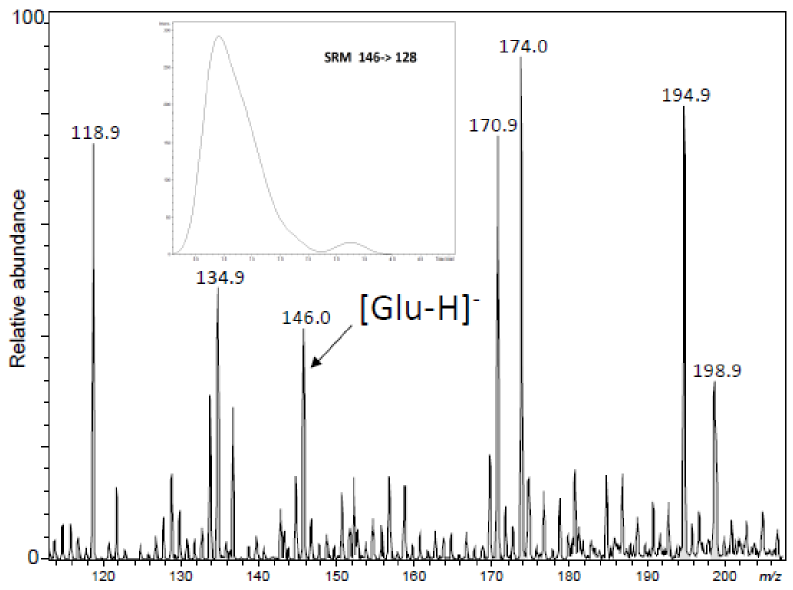

B

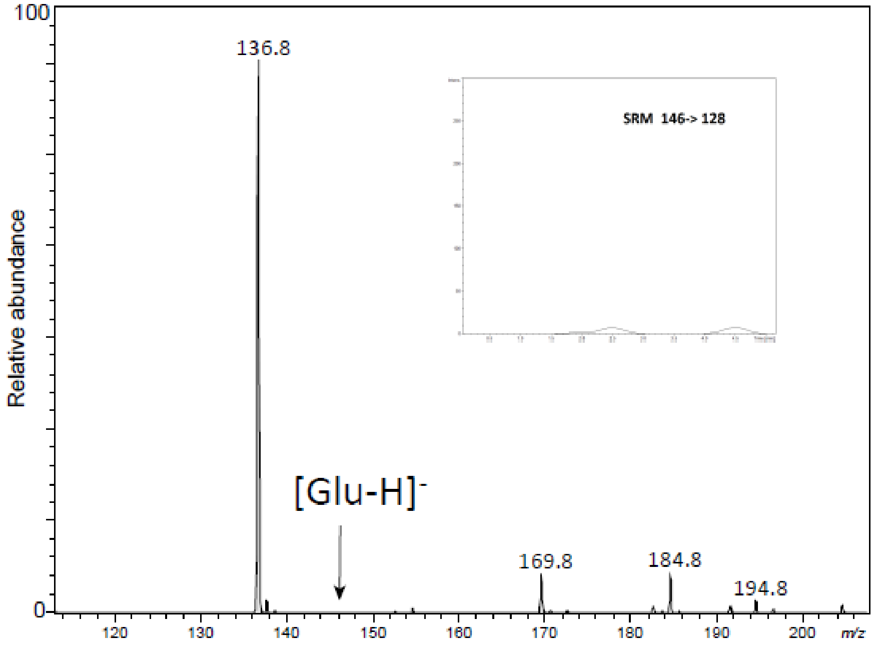
100
136.8
SRM 146-> 128
Relative abundance
[Glu-H]-
169.8
184.8
194.8
0
120
130
140
150
160
170
180
190
200
m/z

C

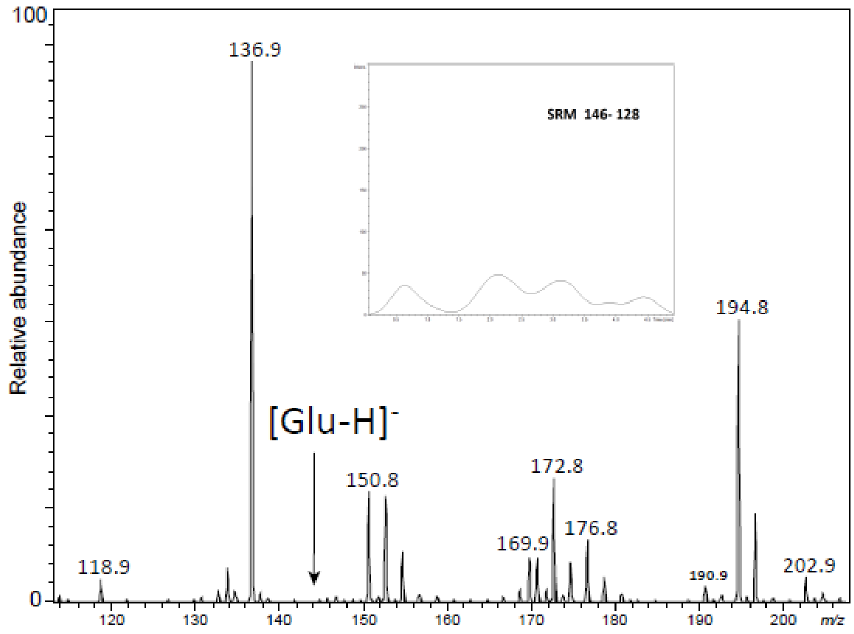
100
136.9
SRM 146- 128
Relative abundance
194.8
[Glu-H]-
150.8
172.8
169.9
176.8
118.9
190.9
202.9
0
120
130
140
150
160
170
180
190
200
m/z

Supplementary Figure 21 | Contribution of intracellular asymmetry to estimation of mass doubling time.

A steady state culture of *E. coli* was labeled for different periods with 90% ^{15}N and the mass doubling time then estimated for each cell and plotted against the imaginary 'mass doubling time' estimated for the half of that cell with the higher incorporation (black squares) and the lower incorporation (orange triangles). (**A**) 2 min (the inset is a zoom of the region of the 40–60 min mass doubling times). (**B**) 4 min. (**C**) 8 min. (**D**) 16 min. (**E**) 32 min. (**F**) 64 min. (**G**) 128 min (the inset is a zoom of the region of the 60–80 min mass doubling times).

A

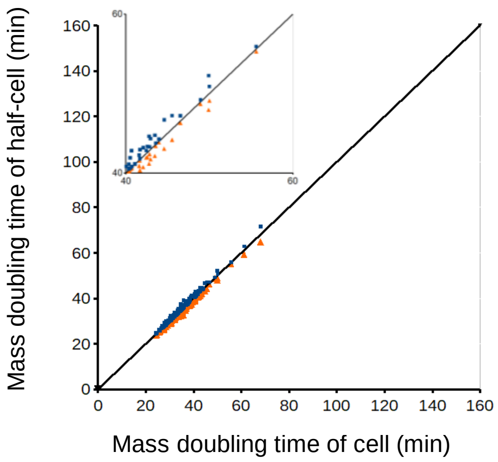

B

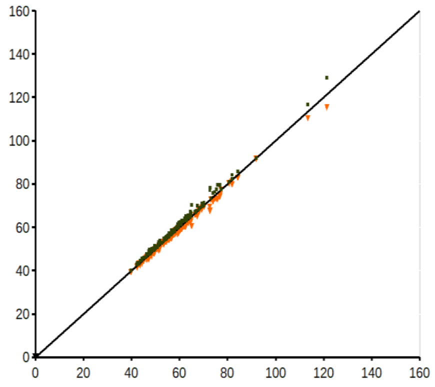

C

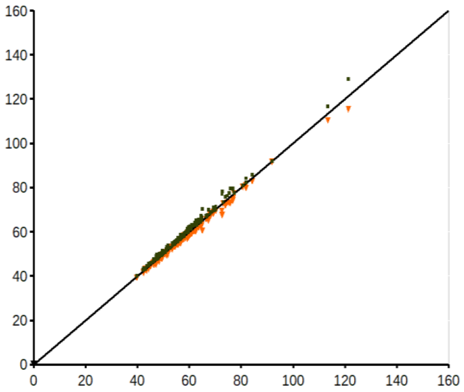

0
20
40
60
80
100
120
140
160

D

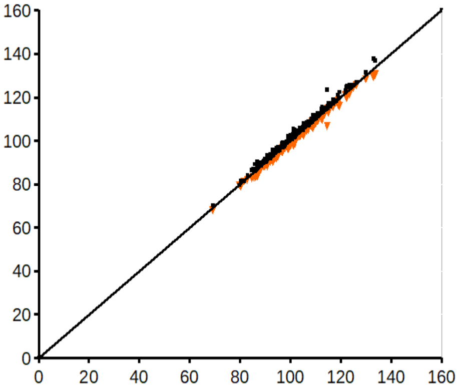

0
20
40
60
80
100
120
140
160

E

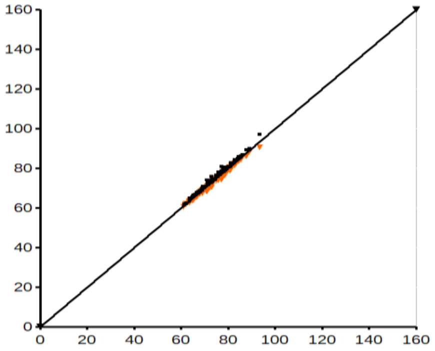

0
20
40
60
80
100
120
140
160

F

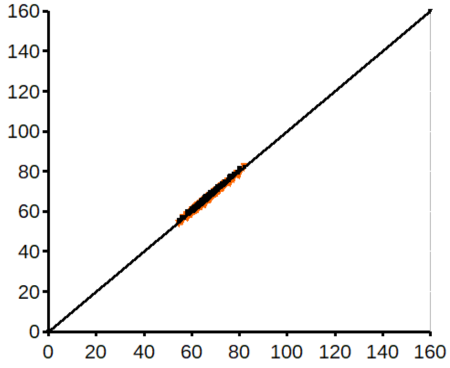
160
140
120
100
80
60
40
20
0
0
20
40
60
80
100
120
140
160

G

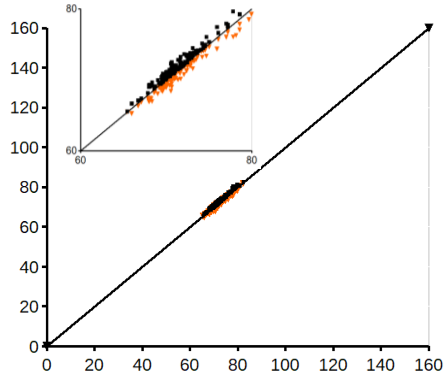
160
140
120
100
80
60
40
20
0
0
20
40
60
80
100
120
140
160

Supplementary Figure 22 | Growth rate does not affect cell size. The size distribution is as expected for a bacterial culture in steady-state growth. Moreover, the size distributions of both the fastest growing cells and the slowest growing cells are the same as the overall size distribution. Cell size was estimated as the number of pixels. All cells (blue), the 10% of cells growing fastest (orange), and the 10% of cells growing slowest (green).

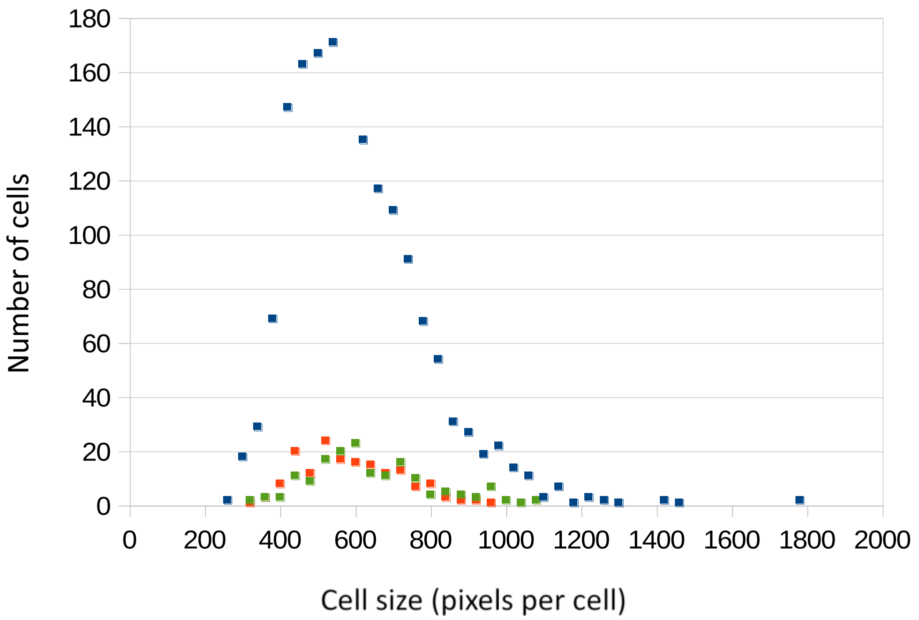

Supplementary Figure 23 │ Characterization of populations of *E. coli* by growth rate and asymmetry indices. The asymmetry index, I_a, plotted against the growth rate index, I_g, for different labeling periods: (**A**) 32 min. (**B**) 64 min. (**C**) 128 min.

A

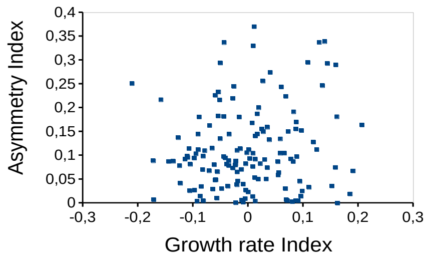

B

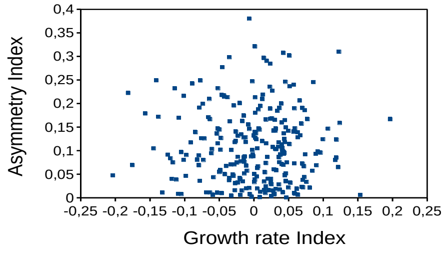

C

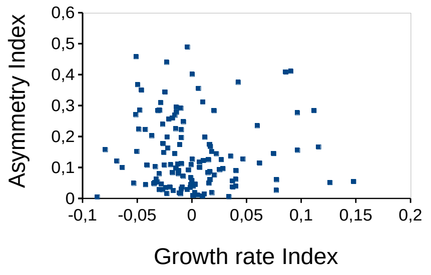

Supplementary Table 1 | Consistency of estimated mass doubling times in *E. coli*. After 64 min labeling and analysis as above, the mass doubling times for seven bacteria were estimated using equation (S7) from each of seven serial sputtered sections.

Bacterium	Area pixels	section 1 $^{15}N/(^{15}N+^{14}N)$	section 2 $^{15}N/(^{15}N+^{14}N)$	section 3 $^{15}N/(^{15}N+^{14}N)$	section 4 $^{15}N/(^{15}N+^{14}N)$	section 5 $^{15}N/(^{15}N+^{14}N)$	section 6 $^{15}N/(^{15}N+^{14}N)$	section 7 $^{15}N/(^{15}N+^{14}N)$
1	836	0.40616	0.41074	0.41418	0.41752	0.41714	0.41715	0.41715
2	1424	0.43029	0.43538	0.43837	0.43783	0.44044	0.44075	0.44075
3	970	0.44758	0.45278	0.4537	0.45564	0.4566	0.45887	0.45887
4	1025	0.42768	0.43369	0.43723	0.43616	0.43824	0.43748	0.43748
5	1049	0.45606	0.46398	0.46625	0.46887	0.47078	0.47373	0.47373
6	955	0.42521	0.43811	0.4424	0.44361	0.4451	0.44577	0.44577
7	1287	0.43293	0.43774	0.43926	0.44028	0.44398	0.44484	0.44484

Bacterium	Area	section 1 Est. gen. time	section 2 Est. gen. time	section 3 Est. gen. time	section 4 Est. gen. time	section 5 Est. gen. time	section 6 Est. gen. time	section 7 Est. gen. time
1	836	72	71	70	70	70	70	70
2	1424	67	66	65	65	65	64	64
3	970	63	62	62	61	61	61	61
4	1025	67	66	65	65	65	65	65
5	1049	61	60	59	59	58	58	58
6	955	68	65	64	64	64	63	63
7	1287	66	65	65	65	64	64	64

Supplementary Table 2 | Contribution of loss of pool to Coefficients of Variation. The random losses of small molecule pools to different degrees in 200 different bacteria was simulated using a program written in Visual Basic 6. The effects on the CVs and on the apparent mass doubling times of the populations was then calculated for three, initially intact, pool sizes after different labeling periods.

**Labeling period min**	**Mean + %CV for 2% pool**	**Mean + %CV for 3% pool**	**Mean + %CV for 4% pool**
2	78, 20	78, 20	75, 20
4	72, 12	74, 14	72, 15
8	68, 6.2	69, 7.8	69, 9.3
16	66, 3.0	67, 3.9	67, 4.9
32	65, 1.5	65, 1.9	65, 2.4
64	64, 0.72	65, 0.94	65, 1.2
128	64, 0.36	64, 0.47	64, 0.58

Supplementary Table 3 | Estimation of percentage of dividing cells of *E. coli* after different labeling periods. The only cells with an estimated growth rate that could be affected by the asymmetric inheritance of labeled/labeled material are those born during the labeling period. The fraction of a theoretical population of identical cells comprising those born during the labeling period was estimated for each labeling period (**Material and Methods**).

Labeling period (min)	Labeled divided cell % of total population
2	4
4	8
8	17
16	32
32	59
64	100
128	100

Supplementary Table 4 | Quantification of growth rate diversity in an *E. coli* population for the longer periods of labeling. The growth rate index, I_g, is defined as (the signed value of the difference between the estimated growth rate of the bacterium and the growth rate of the sample population)/the growth rate of the sample population; by definition, the sample population has a mean I_g of zero and therefore the standard deviation of I_g equals the coefficient of variation of the mass doubling times (CV). The asymmetry index, I_a, is defined as the absolute value of the fraction of pixels below the cell's median isotope ratio in one half-cell minus the fraction of pixels below this median in the other half-cell. The results are for the A series.

Labeling time/number of bacteria	SD of I_g = CV	Mean(I_a) ± SD of I_a
32 min/142	0.084	0.111±0.086
64 min/258	0.063	0.109±0.077
128 min/127	0.042	0.145±0.115
